# Highly Oriented Bio‐Mimetic Hydrogels by Calendering

**DOI:** 10.1002/advs.202504778

**Published:** 2025-06-19

**Authors:** Zhanqi Liu, Yuqing Wang, Haidi Wu, Huamin Li, Longcheng Tang, Guo Wang, Daxin Zhang, Jianping Yin, Yinggang Miao, Yongqian Shi, Pingan Song, An Xie, Xuewu Huang, Wancheng Gu, Yiu Wing Mai, Jiefeng Gao

**Affiliations:** ^1^ School of Chemistry and Chemical Engineering Yangzhou University No 180, Road Siwangting Yangzhou Jiangsu 225002 China; ^2^ Key Laboratory of Organosilicon Chemistry and Material Technology of Ministry of Education College of Material Chemistry and Chemical Engineering Hangzhou Normal University Hangzhou 311121 China; ^3^ Shanxi Key Laboratory of Impact Dynamics and its Engineering Application School of Aeronautics Northwestern Polytechnical University Xi'an 710072 China; ^4^ College of Environment and Safety Engineering Fuzhou University Fuzhou 350116 China; ^5^ Centre for Future Materials University of Southern Queensland Springfield Campus Southern Queensland QLD 4300 Australia; ^6^ Testing Center Yangzhou University Yangzhou Jiangsu 225002 China; ^7^ Department of Mechanical Engineering The Hong Kong Polytechnic University Hung Hom, Kowloon Hong Kong 999077 China

**Keywords:** anisotropic hydrogels, calendering, fatigue resistance, mechanical properties

## Abstract

Anisotropic hydrogels are promising candidates as load‐bearing materials for tissue engineering, while huge challenges remain in exploring effective and scalable methods for the preparation of anisotropic hydrogels with simultaneous high tensile strength, large toughness, good fracture strain, excellent fatigue and swelling resistances. Inspired by the brick‐and‐mortar layered structure of nacre and the hierarchical fibril strucure of soft tissues (e.g., tendon and ligament), a facile organogel‐assissted calendering strategy is reported to design anisotropic hydrogels with a highly oriented and dense fiber lamellar strucure. The synergy of shearing and annealing promotes macromolecular chain alignment and crystallinity along the calendering direction while forming a nacre‐like lamellar morphology in the thickness direction. The tensile strength, elastic modulus, toughness and fracture energy of the anisotropic hydrogels can reach as high as 41.0 ± 6.4 MPa, 67.0 ± 5.1 MPa, 46.2 ± 3.3 MJ m^−3^, and 62.20 ± 8.55 kJ m^−2^, respectively. More importantly, the hydrogels show excellent crack growth and swelling resistances with the fatigue threshold increased to 2170 J m^−2^. This study provides a promising approach for fabrication of large‐sized biomimetic anisotropic hydrogels with outstanding mechanical properties for biomedical and engineering applications.

## Graphical abstract

A facile and scalable organogel‐assisted calendering strategy is proposed to prepare bio‐mimetic anisotropic polyvinyl alcohol (PVA) hydrogels. By employing a combination of shearing and annealing during calendering, PVA chains are aligned in the calendering direction, while the compression in thickness facilitates the formation of a nacre‐like laminated structure with highly oriented fibers. These anisotropic hydrogels exhibit exceptional mechanical properties.

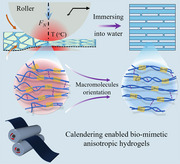

## 1 Introduction

Facile structure design aiming to achieve biological organization is critical for the development of advanced biomimetic materials. Hydrogels, as unique flexible polymeric materials, possess many advantages, such as water‐rich 3D networks, good biocompatibility, and stimulus‐responsive behavior. The demand of high‐performance hydrogels as load‐bearing components is increasing in the fields of implantable devices, soft tissues, etc.^[^ ^1^, ^2^, ^3^, ^4^, ^5^, ^6^, ^7^, ^8^ ^]^ However, because of the plasticizing effect of water and lack of hierarchical structures, hydrogels have difficulties to replicate characteristic properties of natural products.^[^ ^9^ ^]^ For instance, nacres, tendons, spider silk, and heart valves exhibit excellent tear resistance and long‐term stability, and it is clear that the mechanical properties of these natural products with complex multi‐layered/orientated structures far exceed those that can be obtained from simple mixtures of their finite constituents.^[^ ^10^, ^11^, ^12^, ^13^ ^]^ Therefore, structure design is one of the most important and fundamental topics in the scientific study of hydrogels and has been extensively explored in the past decade. For instance, freeze‐thawing, solvent exchange, salting‐out, annealing, and other methods are often used to obtain isotropic hydrogels.^[^ ^14^, ^15^, ^16^, ^17^, ^18^, ^19^, ^20^, ^21^ ^]^ Even though the mechanical properties of hydrogels are optimized, polymer networks with random orientations still constrain further enhancement of their fatigue resistance. Thus, anisotropic hydrogels with highly ordered fibril structures akin to those of natural products have been prepared by freeze‐casting, mechanical training, pre‐stretching, drying in confined conditions (DCC), and flow‐induced orientation.^[^ ^11^, ^22^, ^23^, ^24^, ^25^, ^26^, ^27^ ^]^ Hydrogels fabricated via freeze‐casting typically lack macromolecular orientation, as their structure is dominated by ice‐templated pore architectures rather than aligned polymer chains. This disordered network compromises optical properties like transparency, as well as mechanical properties such as strength, toughness, and energy dissipation. To enhance these mechanical properties, researchers often employ post‐treatments such as salting‐out, which increases cross‐link density and facilitates molecular chain rearrangement. But the strong anisotropic hydrogels containing salts are also unsuitable for applications in aqueous environment since the ions readily diffuse outside the hydrogels. As for the stretching‐enabled anisotropic hydrogels, it often fails to fully activate macromolecular motion or optimize chain conformations due to viscoelastic hysteresis and the constraints imposed by the existing network. This limits the extent to which mechanical performance can be improved. Hence, it is highly desirable but still challenging to explore new yet effective processing techniques for the fabrication of large‐sized anisotropic hydorgels with simultaneus enhancment of tensile strengh, toughness, fatigue, and swelling resistances.

Herein, we proposed a facile and scalable organogel‐assissted calendering strategy to prepare anisotropic polyvinyl alcohol (PVA) hydrogels. Through the synergy of shearing and annealing actions during calendering, PVA chains were oriented along the calendering direction, while the compression in the thickness direction promoted the fomation of a nacre‐like laminated structure comprising highly oriented fibers. These biomimetic anisotropic hydrogels displayed remarkable mechanical properties with tensile strength, toughness, and elastic modulus up to 41.0 ± 6.4 MPa, 46 ± 3.3 MJ m^−3^ and 67.0 ± 5.1 MPa, respectively. More importantly, the hydrogels possessed fatigue crack growth thresholds as high as 2170 J m^−2^. This work will open up a new methodology for large‐scale manufacture of high‐performance anisotropic hydrogels.

## 2 Results and Discussion

**Figure 1:**
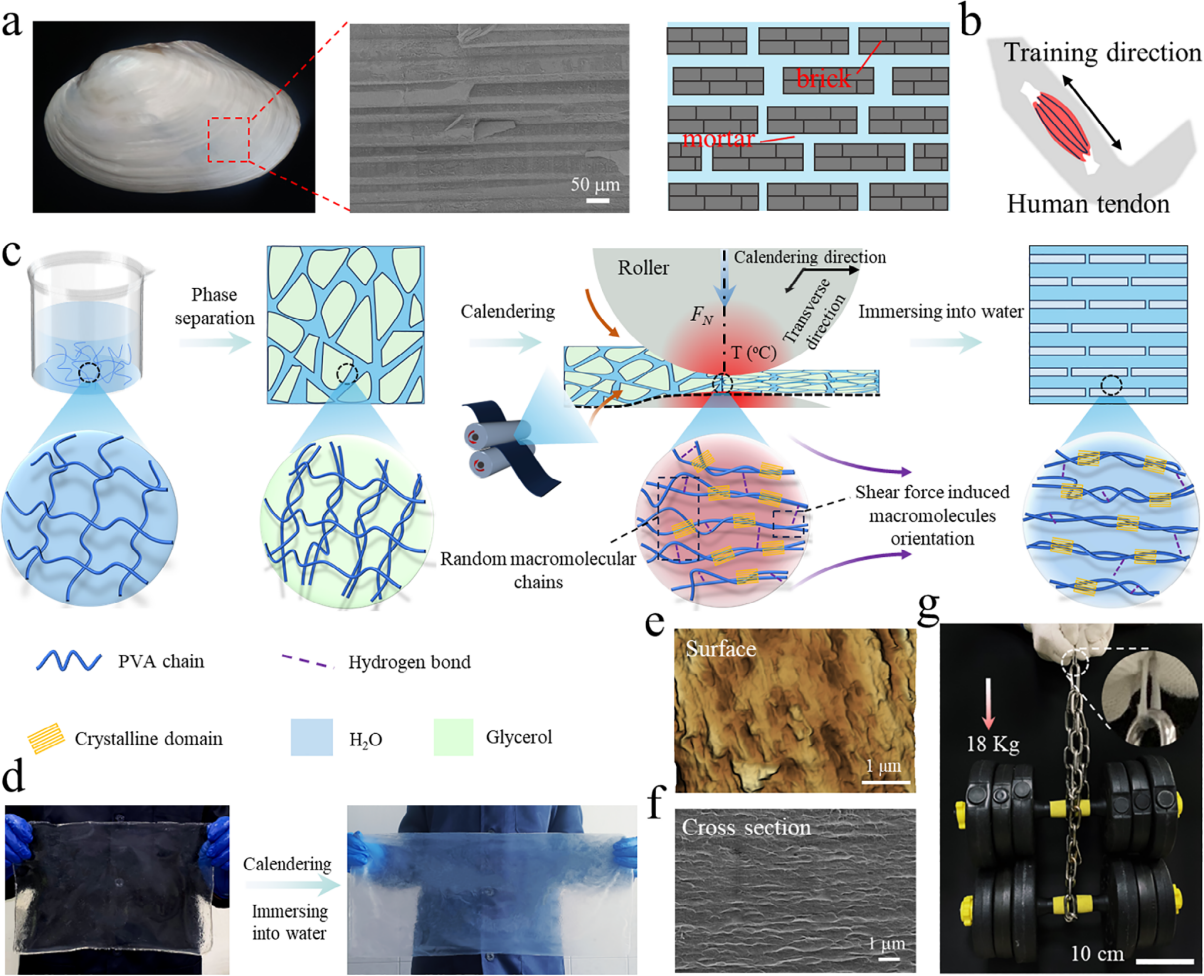
Preparation of strong and tough anisotropic hydrogels. a) Digital photo, SEM image, and schematic of natural nacre, and b) schematic diagram of human tendon. c) Schematic illustration for the fabrication of biomimetic hydrogels by organogel‐assissted calendering. d) Photos of large‐sized PVA organogel and hydrogel. e) AFM and f) SEM images of AC‐120‐0.18. g) Photo showing the remarkable mechanical properties of the hydrogel along the calendering direction by sustaining two dumbbells (18 kg) with over 24000 times its own weight.

Nacre, which consists only of brittle aragonite flakes and low‐strength organic biopolymers, has achieved an excellent combination of apparently contradictory mechanical properties such as strength and fracture energy because of the unique brick‐and‐mortar structure (**Figure** **1**a). The aragonite flakes (“brick”) endow nacre with high strength and high elastic modulus, while the organic biopolymers (“mortar”) are responsile for the outstanding fracture resistance.^[^ ^12^, ^28^ ^]^ It is also critical for the synthetic materials to possess excellent fatigue cyclic crack growth resistance to ensure their long‐term stability. As shown in Figure 1b, soft issues such as tendons and ligaments usually display anisotropic fibrous structure. Biopolymers, such as collagen, are secreted from the cells and assembled into fibrils, which can then be aligned to form dense hierarchical structures from nanoscopic to macroscopic length scales. When the micro‐crack is transverse to the direction of fiber alignment, these soft tissues can effectively resist external impact and diffuse the stress, exhibiting excellent fracture resistance due to the rigidity of the aligned fibers and the rich physical interactions between the macromolecular chains.

Inspired by nacre and soft tissues, we have designed anisotropic PVA hydrogels with excellent mechancial properties by calendering which combines shearing and wet‐annealing. As shown in Figure 1c, the uniform PVA/water solution is first immersed into glycerol for solvent exchange. Since glycerol is a poor solvent for PVA but misible with water, the water in the PVA solution is gradually replaced by glycerol, during which phase separation of PVA occurrs. Thus, PVA macromolecules aggreatge and futher gelate to form PVA/glycerol organogel. Notably, the obtained organogel shows good mechanical properties and thermal stability owing to the high boiling point of glycerol, which ensures the structure integrity of the organogel during calendering that provides shear forces and annealing. The glycerol gel facilitates dynamic rearrangement of PVA chains through its plasticizing effect, while the gradual volatilization of glycerol maintains the orientation stability of the macromolecular chains. This dual mechanism ensures thermal equilibrium during the annealing process, ultimately leading to significant improvement in crystallinity. Decreasing the roll gap increases significantly the shear forces, leading to orientation of macromolecular chains along the calendering direction. In addition, annealing during calendering also facilitates the adjustment of macromolecular conformation and hence the formation of anisotropic structures. Also, calendering can promote the crystallization of the macromolecular chains. It is noted that the anisotropic structure with a densified polymer network and crystalline domains are maintained after the calendered organogel is immersed into deionized water to obtain the hydrogel. For ease of discussion, hereafter, the hydrogel fabricated by the two‐step solvent exchange without calendering is designated TSE, and the anisotropic hydrogels by calendering, AC‐x‐y or AT‐x‐y, where x and y refer to the calendering temperature (°C) and roll gap (mm), respectively. C and T stand for the calendering (macromolecule alignment direction) and transverse directions, respectively. Furthermore, isotropic hydrogels prepared by calendering are denoted by C‐x‐y and T‐x‐y. Figure 1d displays large‐sized anisotropic organogel and hydrogel (AC‐120‐0.6) with good transparency (Figures S1 and S2, Supporting Information). The AFM image of the hydrogel (AC‐120‐0.18) surface displays a clear sheet‐like structure (Figure 1e); the lamellar structure comprising orientated fiber bundles is found on the cross‐section of the hydrogel along the calendering direction (Figure 1f), which confirm the nacre‐ and soft tissues‐ inspired biomimetic structure has been successfully constructed. The anisotropic hydrogels show impressive mechanical properties, and a thin hydrogel (AC‐120‐0.18) can even withstand loads exceeding 24000 times its own weight (Figure 1g).

**Figure 2:**
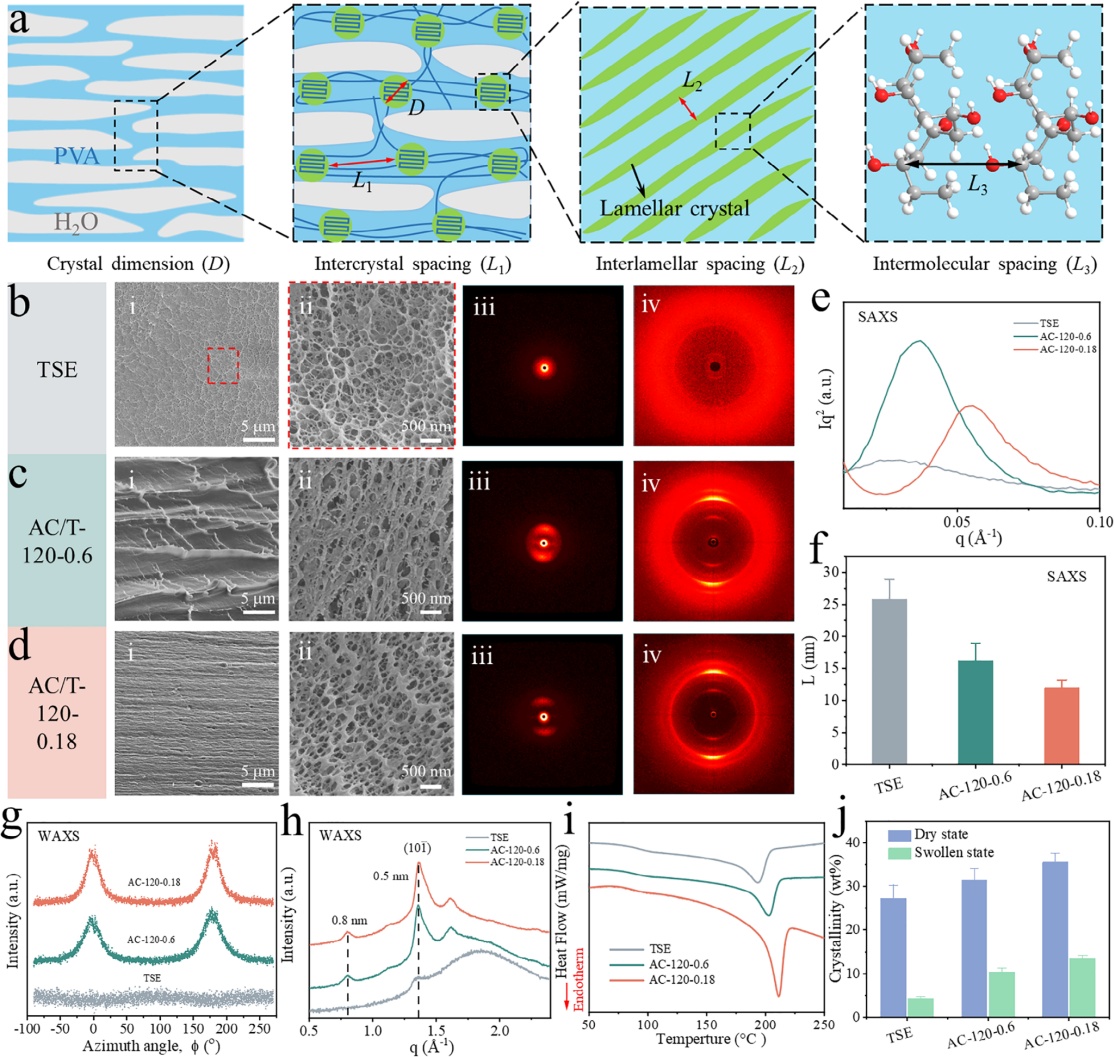
The morphology and crystalline structure of different hydrogels. a) Schematic crystalline structure for the anisotropic hydrogels. SEM images in i) calendering‐ and ii) transverse‐direction, and iii) Small‐angle X‐ray scattering (SAXS) and iv) Wide‐angle X‐ray scattering (WAXS) patterns of b) TSE, c) AC‐120‐0.6, and d) AC‐120‐0.18. e) SAXS profiles and f) calculated average distance between adjacent nanocrystalline domains. WAXS profiles of intensity versus g) azimuthally and h) scattering vector. i) Differential scanning calorimetry (DSC) curves and j) calculated crystallinity of hydrogels in dry and swollen states.

Organogel‐assisted calendering created densified anisotropic structure with abundant crystalline domains in the hydrogels, and the detailed morphology and crystal structures are schematically shown in **Figure** **2**a. *D* refers to the crystal size while *L*_1_ represents the distance between the adjacent crystal domains. Each crystal domain comprises multi‐layered lamellar crystals with an interlamellar spacing of *L*_2_. In each lamella, the intermolecular spacing is given by *L*_3_. A series of characterizations were performed to identify the anisotropic structure and crystal structure. As shown from the SEM images in Figure 2b‐i and b‐ii, TSE exhibits a homogeneous and disordered porous structure with isotropic characteristics. However, no pores are found for AC‐120‐0.6 in the calendering direction. Instead, a laminar structure with aligned macro‐grooves is found; and the cross‐section in the transverse direction displays a compressed porous structure (Figure 2c‐i and c‐ii) when compared to that of TSE. With the decrease of the roll gap (i.e., increase of shearing force), AC‐120‐0.18 shows a more obvious nanofiber orientation structure in the calendering direction, and the interlamellar spacing is a few hundreds of nanometers, much smaller than that of AC‐120‐0.6, and the pore size in the transverse direction also decreases (Figure 2d‐i and d‐ii; Figure S3, Supporting Information). The morphological difference between the two directions is because the initially isotropic porous structure undergoes fusion and reorganization along the calendering direction, resulting in a marked reduction or even complete disappearance of pores. In contrast, the transverse direction (perpendicular to the calendering direction) retains a more intact porous morphology due to limited uniaxial stretching, thereby revealing pronounced anisotropy in SEM observations. The above results indicate that the narrow roll gap at a high temperature can provide strong shearing force and endow the hydrogel with a denser and more ordered network structure. The hot shearing‐induced orientation structure of the hydrogels is also clearly shown in optical microscopy, and the surface of AC‐120‐0.18 has an ultra‐regular alignment of the fiber bundles (Figure S4, Supporting Information). The morphology of the hydrogels confirms that highly aligned fiber bundles are stacked together to form an ordered and dense lamellar structure because of the calendering induced thinning of the organogels. This unique structure combining the characteristics of nacre and soft issues demonstrate the feasibility of calendering as a facile and efficient approach to prepare biomimetic anisotropic hydrogels.

To study the orientation and crystallinity of the PVA chains, in situ small‐angle X‐ray scattering (SAXS) and wide‐angle X‐ray scattering (WAXS) tests were performed. As shown in Figure 2a‐iii and a‐iv, the 2D SAXS and WAXS patterns of TSE showed nearly round and uniform intensity rings at the whole azimuthal angles, indicating an isotropic structure. The SAXS patterns of both AC‐120‐0.6 and AC‐120‐0.18 displayed ellipsoidal 2D patterns with bright arcs, and their WAXS patterns also showed the same variations as SAXS (Figure 2b‐iii, iv and 2c‐iii, iv), confirming evident anisotropic structures. These results reveal that the high orientation of PVA chains promotes the formation of fiber bundles in the calendering direction. Further, the scattering intensity *I* versus scattering vector *q* was measured to quantify the evolution of the crystalline domains during calendering. To clearly identify the location of the peak intensity, we corrected the intensity by multiplying the scattering intensity *I* with the square of the scattering vector (i.e., *Iq* ^2^). From the Bragg equation (*L* = 2*π*/*q*_max_), *L*_1_ could be estimated from the scattering vector at the peak position (Figure 2e). For TSE, the average distance between adjacent crystalline domains was 25.7 ± 3.2 nm, while the distance decreased to 16.1 ± 2.8 and 12.0 ± 1.2 nm for AC‐120‐0.6 and AC‐120‐0.18, respectively (Figure 2f). Furthermore, *D* for TSE, AC‐120‐0.6, and AC‐120‐0.18 were calculated to be ≈6.8 nm for all samples. Coupled with the decrease in the average distance between adjacent crystalline domains, it can be concluded that the enhancement of crystallinity is owing more to the crystalline domains than the increase of crystal size. Subsequently, based on the empirical equation (*Π* = (180− FWHM) /180), the orientation degree (*Π*) of PVA chains were obtained from the plot of the intensity versus the azimuth angle (Figure 2g). For TSE, no peak was found, which implied negligible orientation of PVA chains. For AC‐120‐0.6 and AC‐120‐0.18, the orientation degree of PVA chains were 0.80 and 0.85, respectively, confirming the presence of highly oriented crystallites in the hydrogels. As displayed in Figure 2h, *L*_3_ of the hydrogels, which corresponds to the typical reflection plane of (10$\bar{1}$) of semi‐crystalline PVA,^[^ ^29^, ^30^, ^31^ ^]^ is 0.5 nm. Besides, small peaks at 0.8 nm (*L*_2_) were found for the anisotropic hydrogels, indicating that compared to TSE, calendering gave more pronounced crystallization of PVA chains for AC‐120‐0.6 and AC‐120‐0.18.

Differential scanning calorimetry (DSC) tests were conducted to further quantify the crystallinity of the hydrogels. As shown in Figure S5 (Supporting Information), the melting point of TSE, AC‐120‐0.6 and AC‐120‐0.18 are 200.2 ± 5.8, 207.6 ± 4.0, and 211.7 ± 0.8 °C, respectively, and the increased melting point implied more crystalline domains in the hydrogels. According to the enthalpies and Equation (S7, Supporting Information), the crystallinity of TSE, AC‐120‐0.6, and AC‐120‐0.18 in the dry state was calculated to be 27.2 ± 3.1%, 31.5 ± 2.6%, and 35.6 ± 2.1%, respectively, and the corresponding crystallinity in the swollen state based on Equation (S8, Supporting Information) was 4.3 ± 0.5%, 10.4 ± 0.9%, and 13.4 ± 0.8%, respectively, (Figure 2i,j). AC‐120‐0.18 showed the highest crystallinity, which indicated that strong shearing during calendering can refine and densify the arrangemnt of the PVA chains and promote crystallinity.

Further to the crystalline behavior, calendering also infuences the intermolecular interactions. As shown in Figure S6 (Supporting Information), the absorption peaks of the stretching vibration of the hydroxyl group (─OH) of PVA hydrogels are noticed in the attenuated total reflection Fourier transform infrared (ATR‐FTIR) spectra. It is found that the characterisitc peak was markedly shifted to the lower wave‐number regions for the anisotropic hydrogels. For example, the peak shifted from 3332 for TSE to 3313 and 3303 cm^−1^ for AC‐120‐0.6 and AC‐120‐0.18, respectively. These results confirm enhanced hydrogen bonding between the PVA chains.^[^ ^32^ ^]^ Meantime, the characteristic peak located at 1142 cm^−1^ corresponds to the C─O vibration of PVA that is related to the formation of crystalline domains;^[^ ^14^ ^]^ and AC‐120‐0.18 exhibits the maximum peak intensity, indicating the formation of more crystalline domains in the hydrogels.

**Figure 3:**
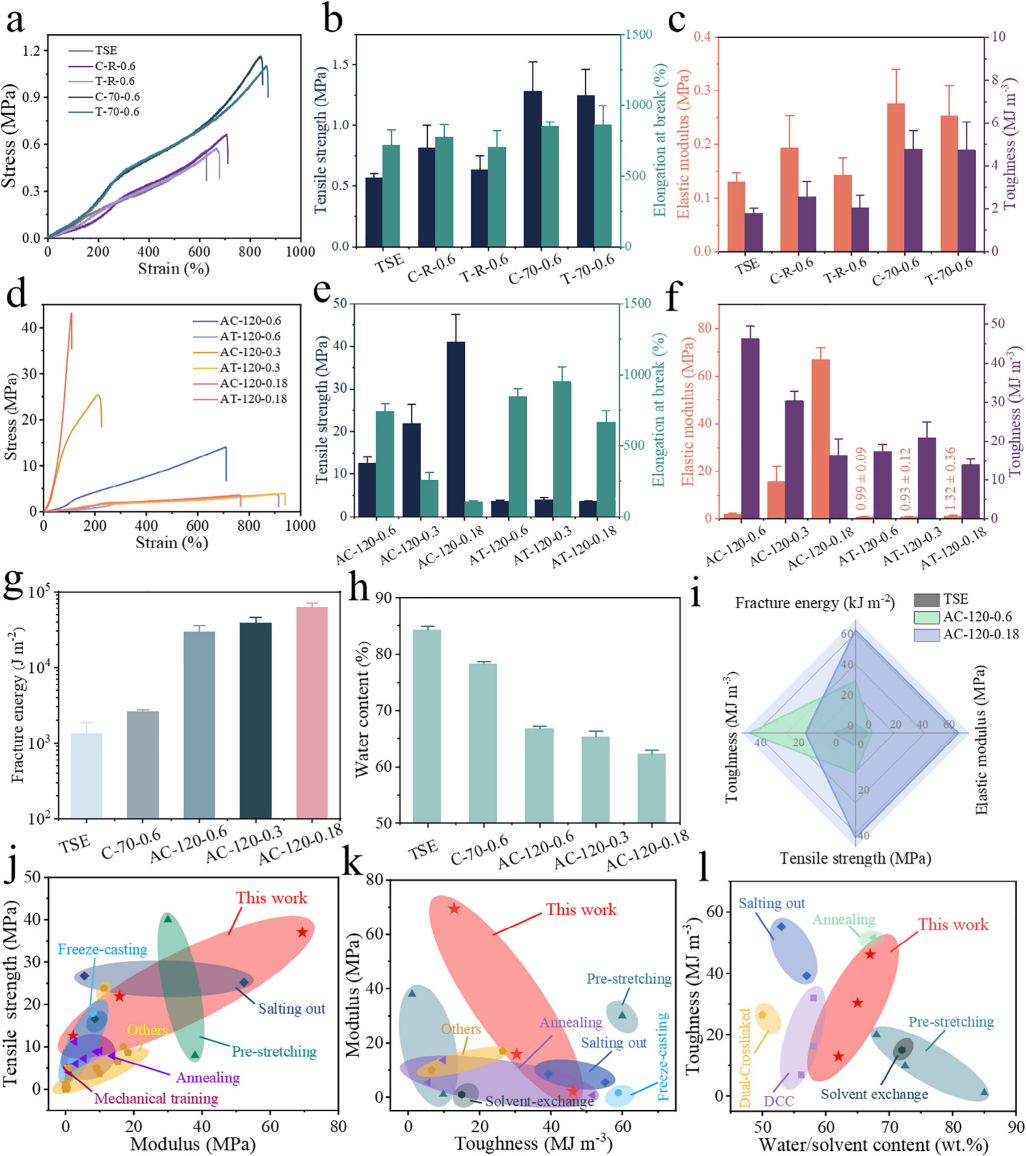
Mechanical properties of PVA hydrogels. Typical tensile stress‐strain curves of a) isotropic and d) anisotropic hydrogels and their corresponding mechanical properties, including b,e) tensile strength and elongation at break, and c,f) elastic modulus and toughness. g) Fracture energy and h) water content of different hydrogels. i) Comparison of mechanical properties of TSE, AC‐120‐0.6, and AC‐120‐0.18. Comparison of j) tensile strength versus elastic modulus, k) elastic modulus versus toughness, and l) toughness versus water content of the anisotropic hydrogels in this study and other reported hydrogels.

**Figure** **3**a–c shows typical stress‐strain curves and a summary of the mechanical properties of the “control” hydrogel samples. The tensile strength, elongation at break, elastic modulus, and toughness of TSE were 0.57 ± 0.03, 718 ± 109%, 0.13 ± 0.02, and 1.78 ± 0.24 MJ m^−3^, respectively. The mechanical properties of C‐R‐0.6 and T‐R‐0.6 display only a slight increase (R refers to room temperature). However, when the calendering temperature is increased to 70 °C, C‐70‐0.6 and T‐70‐0.6 possess tensile strength of 1.28 ± 0.24 and 1.25 ± 0.22 MPa, elastic modulus, 0.28 ± 0.06 and 0.25 ± 0.06 MPa, and elongation at break of 851 ± 33% and 860 ± 137%, respectively. Their toughness is accordingly enhanced to 4.79 ± 0.06 and 4.75 ± 1.30 MJ m^−3^, respectively. Compared to TSE, both C‐70‐0.6 and T‐70‐0.6 show large increases in mechanical properties, however, no anisotropy in both the calendering and transverse directions is found. Despite the presence of a strong shearing force, it is difficult for PVA chains to effectively adjust their conformation and arrangement to achieve alignment in the glycerol gel at a relatively low temperature, since strong intermolecular interactions prevent the macromolecular chain movement to a great extent. However, when the calendering temperature rose to 120 °C, the tensile strength, elastic modulus, and toughness of AC‐120‐0.6 were all significantly improved to 12.56 ± 1.40 MPa, 2.16 ± 0.32 MPa, and 46.19 ± 3.28 MJ m^−3^, respectively, much higher than those of AT‐120‐0.6, exhibiting evident anisotropy in mechanical properties (Figure 3d–f). These results indicate that the increase of calendering temperature can provide sufficient energy to overcome the resistance of macromolecule motions and hence facilitate the orientation of macromolecular chains along the calendering direction, *viz*., the shearing force direction. Once the organogel was cooled and transformed to the hydrogel via solvent exchange, the macromolecule chain alignment and anisotropic structure are preserved, as can be verified from the optical and SEM images (Figure S4, Supporting Information; Figure 2b,c). To explore the effect of shearing force on the mechanical properties of hydrogels, the roll gap was further decreased. As a result, the tensile strength of AC‐120‐0.3 and AC‐120‐0.18 was improved to 21.93 ± 4.40 and 41.03 ± 6.41 MPa, respectively, with corresponding elongation‐at‐break of 260 ± 50% and 107 ± 8% as well as toughness of 30.38 ± 2.38 and 16.36 ± 4.26 MJ m^−3^. Also, AC‐120‐0.18 displayed extremely high rigidity with an elastic modulus of 66.98 ± 5.07 MPa. When the roll gap was decreased, the generated shear force increased significantly, and the tensile strength and elastic modulus of the hydrogels were enhanced accordingly, while the elongation‐at‐break decreased as the highly stretched macromolecule chains lack large deformation capability. Especially, the mechanical properties of the anisotropic hydrogels in the transverse direction varied little even though the roll gap became narrower. For example, AT‐120‐0.6, AT‐120‐0.3, and AT‐120‐0.18 possessed tensile strengths of 3.75 ± 0.23, 4.04 ± 0.56, and 3.65 ± 0.11 MPa, respectively, and the corresponding elastic moduli were 0.99 ± 0.09, 0.93 ± 0.12, and 1.32 ± 0.36 MPa.

As shown in Figure 3g and Figure S7 (Supporting Information). The fracture energy of TSE was only 1.32 ± 0.54 kJ m^−2^, and AC‐120‐0.18 showed a maximum fracture energy of 62.20 ± 8.55 kJ m^−2^, ≈47 times that of TSE. Optical microscopy images revealed that the crack in TSE grew smoothly with a low fracture resistance. However, AC‐120‐0.18 showed a deflected crack (Figure S8, Supporting Information) since the highly aligned fibrous structure was transverse to the crack tip. During stretching, the lamellar structure comprising ordered broken fiber bundles would ensure “ductility” owing to their relative sliding. However, crack deflection occurred at the soft‐hard interface between crystalline and amorphous domains, resulting in a curvilinear crack path and increasing the fracture energy (Figure S9, Supporting Information). Apart from the excellent fracture energy, the water content of all anisotropic hydrogels was maintained > 60 wt.% (Figure 3h), comparable to that of tendons.^[^ ^33^ ^]^

The hydrogels were subjected to cyclic tensile tests to study their physical cross‐linking properties and energy dissipation mechanisms. Figure S10 (Supporting Information) displays loading–unloading curves during cyclic tests at 50% strain for different hydrogels, and all exhibit mechanical hysteresis, i.e., the Mullins effect. The dissipated energies of TSE, AC‐120‐0.6, and AC‐120‐0.18 in the first cycle were 0.60, 19.4, and 1396.6 kJ m^−3^, respectively, and the corresponding hysteresis ratios (the integrated area of the hysteresis loop to that of the loading curve) were 5%, 19%, and 58%, respectively. Clearly, AC‐120‐0.18 dissipated more profound energy. However, after 50 cycles, the residual stress and energy dissipation of all hydrogels remained unchanged, indicating the physical cross‐linking network of the hydrogels was broken to some extent and then rearranged to form a directional structure in the direction of the external force.^[^ ^34^ ^]^ The anisotropic hydrogels exhibit excellent energy dissipation by virtue of their fully physically cross‐linked structure. The oriented PVA chains provide efficient stress transfer along the fiber direction to avoid stress concentration, the dynamic hydrogen bonding network continuously dissipates mechanical energy through reversible fracture and reorganization processes, and the reorganizable crystalline domains can effectively disperse localized stress. This multi‐scale synergistic structural design enables the material to achieve excellent high‐energy dissipation while maintaining integrity.

Rheology tests were conducted to explore the effect of calendering on the physical cross‐linking network of hydrogels. Figure S11 (Supporting Information) displays the variations of storage modulus (*G*′) and loss modulus (*G*″) with time, shear strain and frequency for TSE, C‐70‐0.7, and AC‐120‐0.6. For all the hydrogels, *G*′ is much higher than *G*′′ in the linear viscoelastic region (LVE), which indicates that they have strong elastic properties (Figure S11a–d, Supporting Information). Figure S11b (Supporting Information) shows *G*′ and *G*′′ of the hydrogels with oscillatory strain, and the intersection point of *G*′ and *G*″ is defined as the critical point of gelation (*γ*_c_).^[^ ^35^ ^]^ Compared to AC‐70‐0.6 and TSE, AC‐120‐0.6 possesses the highest *G*′ and *G*″ in LVE, which is attributed to the strong intermolecular interactions that enhance the physical cross‐linking and hinder the movement of the macromolecule chains. When the oscillating strain is larger than 1.4% (γ_c_), the gelation structure of AC‐120‐0.6 is invalidated, and the transformation of hydrogels from elastic solid‐dominated state to viscous fluid‐dominated state is accompanied by the breakdown and reorganization of non‐covalent interactions.^[^ ^36^, ^37^ ^]^ All these results suggest that calendering can modulate the modulus and optimize the mechanical properties of the hydrogels. Figure S11c,d (Supporting Information) displays the frequency scans of hydrogels at 0.1% strain from 0.1 to 100 rad s^−1^. All hydrogels exhibit frequency‐dependent modulus in the full frequency range, displaying viscoelasticity of the hydrogels. *G*′ increases while *G*″ decreases with increasing frequency. Compared to TSE, the loss factor (tan *δ* = *G*″/ *G*′) of C‐70‐0.6 and AC‐120‐0.6 is reduced sharply in the low frequency (0–0.5 rad s^−1^) range, indicating higher viscous dissipation behavior of the hydrogels.^[^ ^36^, ^37^ ^]^

Figure 3i compares the mechanical properties of TSE, AC‐120‐0.6, and AC‐120‐0.18. Anisotropic hydrogels possess much better mechanical properties than TSE. Thus, tensile strength, elastic modulus, toughness, and fracture energy of AC‐120‐0.18 are as high as 41.03 ± 6.41 MPa, 66.98 ± 5.07 MPa, 16.51 ± 2.6 MJ m^−3^, and 62.20 ± 8.55 kJ m^−2^, which are 71, 514, 8, and 46 times higher than the respective properties of TSE. Here, the organogel‐assisted calendering strategy displays some unique advantages for the preparation of super‐strong and tough hydrogels with satisfactory fracture strain, compared with other methods, e.g., pre‐stretching and directional freezing. Figure 3j–l and Table S1 (Supporting Information) summarize the mechanical properties of anisotropic hydrogels studied in this work, and compare them to those of various other hydrogels which are reported in the literature. The anisotropic hydrogel exhibits extraordinarily high strength and elastic modulus, as well as relatively large toughness.

**Figure 4:**
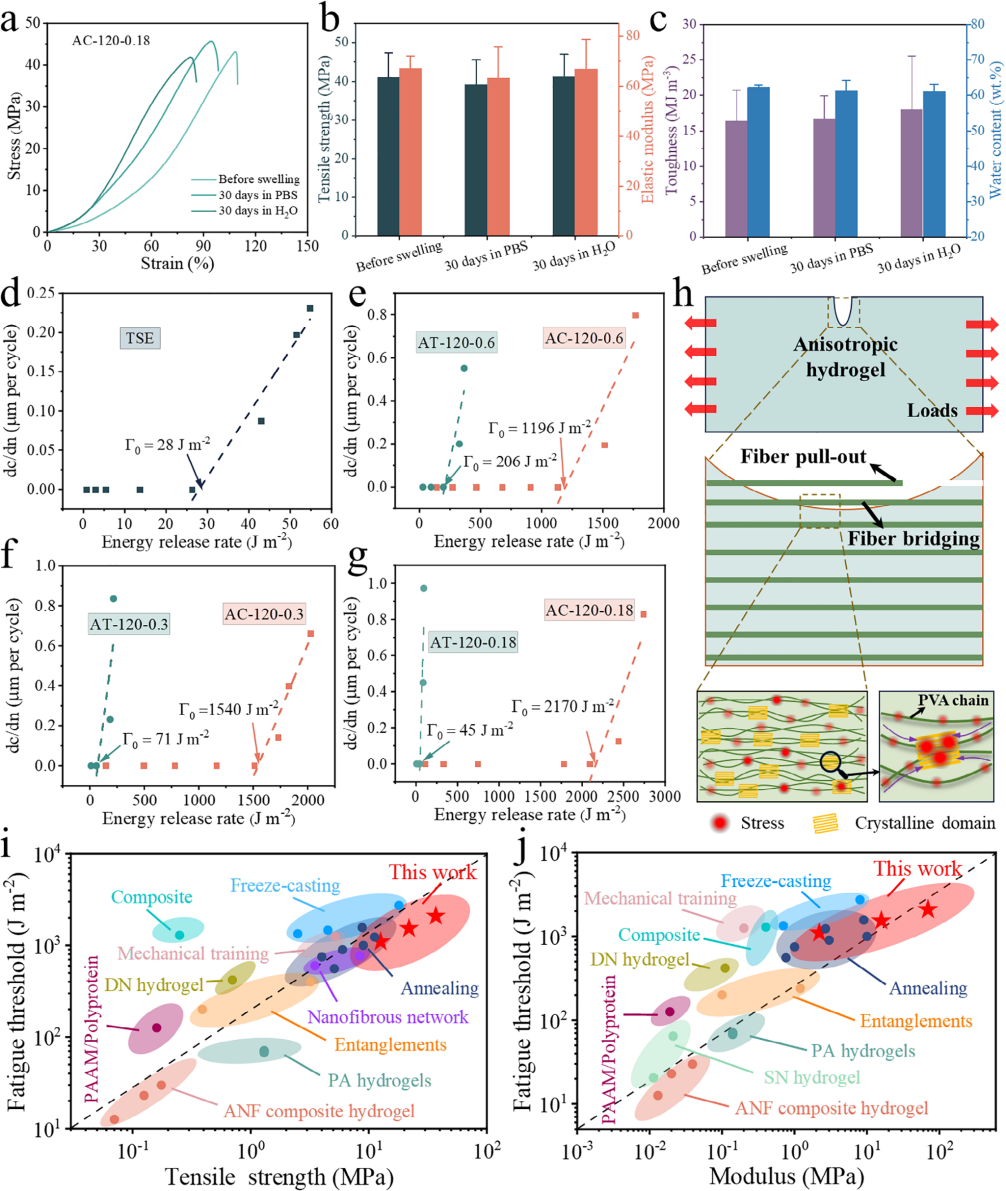
Anti‐swelling and anti‐fatigue properties of the anisotropic hydrogels. a) Typical tensile stress‐strain curves of AC‐120‐0.18 swollen in PBS and water for 0 day and 30 days, and summary of b) tensile strength and elastic modulus, and c) toughness and water content. Crack growth rate (dc/dn) versus applied energy release rate (*G*) and fatigue threshold (*Γ*_0_) for d) TSE, e) AC‐120‐0.6 and AT‐120‐0.6, f) AC‐120‐0.3 and AT‐120‐0.3, and g) AC‐120‐0.18 and AT‐120‐0.18. h) Schematic showing mechanism of fatigue resistance. Fatigue thresholds plotted against i) tensile strength and j) modulus for different hydrogels.

Currently, strong hydrogels with high strength and high modulus are often characterized by high crystallinity but low water content.^[^ ^19^ ^]^ These hydrogels typically absorb water and swell in aqueous environments, resulting in unstable microstructure and mechanical properties, which greatly limit their applications in practical water‐rich situations. As shown in Figure S12 (Supporting Information), AC‐120‐0.18 was immersed in water and phosphate buffered saline (PBS) solution, respectively, for 30 days, and the hydrogel showed little change in both shape and size. Notably, the mechanical properties and water content (**Figure** **4**a–c) of AC‐120‐0.18 remained almost unchanged. For example, the tensile strengths of AC‐120‐0.18 after swelling in PBS and water were 39.2 ± 6.4 and 41.2 ± 5.8 MPa, respectively, and the corresponding elastic modulus were 63.1 ± 12.5 and 66.8 ± 11.8 MPa, similar to the original values. Moreover, all hydrogels retained the water content of ≈62 wt.%. The anisotropic hdyrogels were transformed from calendered organogles through solvent exchange, and the polymeric network had reached an equilibrium state. Moreover, the densified biomimetic fiber laminar structure with abundant crystalline domains could resist the penetration of water, thus endowing the hydrogels with excellent anti‐swelling properties.

A long‐standing challenge in the design of engineering hydrogels is the conflict between strength and modulus versus cyclic fatigue. Fatigue resistance is critical for practical applications as it governs the service life of hydrogels, especially when microcracks or defects are present. In previous studies,^[^ ^11^, ^22^, ^27^ ^]^ freeze‐casting and mechanical training have shown their effectiveness in enhancing crack growth resistance via the introduction of aligned fiber structures, and the fatigue threshold could reach above 1000 J m^−2^. However, the strength and modulus of those hydrogels are not satisfactory, which greatly limit their applications. In this work, the biomimetic anisotropic hydrogels combine high strength, high modulus and superior fatigue resistance. Figure 4d–g shows that single‐edge notch fatigue experiments are used to quantify the fatigue threshold (FT) of different hydrogels. FT of TSE is only 28 J m^−2^ owing to its low crystallinity and loose polymer network. By reducing the roll gap, FT increases in the calendering direction but decreases in the transverse direction (Figure S13, Supporting Information). Specifically, FTs of AC‐120‐0.6, AC‐120‐0.3, and AC‐120‐0.18 are 1196, 1540, and 2170 J m^−2^, respectively, and they decrease markedly to 206, 71, and 45 J m^−2^ for AT‐120‐0.6, AT‐120‐0.3, and AT‐120‐0.18. When compared to that of TSE, FT of AC‐120‐0.18 is increased by ≈2 orders of magnitude. Figure 4h shows the crack resistance mechanisms when a crack spreads from a notch perpendicular to the fiber bundles. The applied loads are transferred to the aligned fiber bundles in the laminate which effectively deter crack growth due to fiber bridging and fiber pull‐out. In addition, the abundant aligned crystalline domains crosslink the macromolecules and hence greatly restrict crack spreading as a result of numerous crack arresting/micro‐pinning effects.^[^ ^11^, ^22^ ^]^ Moreover, the strong hydrogen bonding in the aligned and densely arranged macromolecular chains also contributes to energy dissipation through reversible bond breakage and reformation during deformation. The biomimetic hierarchal structure therefore greatly improves the mechanical properties of the hydrogels. By contrast, if the crack is sited parallel to the direction of fiber alignment, it can propagate easily along the weak interstitial regions between fiber bundles. Higher anisotropy gives fewer entanglements of macromolecules between fibers and hence low resistance for crack growth. Figure 4i,j compares the fatigue resistance of various hydrogels by plotting the fatigue thresholds versus tensile strength and elastic modulus. Clearly, the anisotropic hydrogels in this work exhibit almost the largest tensile strength and modulus as well as maintaining a high fatigue threshold; their fatigue resistance is superior to most of available synthetic hydrogels.

Traditional anisotropic hydrogels are typically fabricated via freeze‐casting or salting‐out. Freeze‐casting aligns macromolecular chains through unidirectional ice crystallization in PVA solutions, but rapid gelation produces weakly crosslinked networks with low hydrogen bond density and crystallinity, limiting fatigue resistance. Salting‐out enhances crystallinity through macromolecular aggregation, yet requires pre‐oriented structures for effectiveness. Conventional approaches combine unidirectional freezing/pre‐stretching with salt immersion, where salting‐out densifies but cannot independently align networks, making properties dependent on initial anisotropy. In contrast to traditional anisotropic hydrogel fabrication methods that rely on tensile forces or unidirectional freezing, hot calendering applies both shear and compressive forces. This process promotes the formation of a nacre‐like lamellar architecture that synergistically combines the brick‐and‐mortar layering of nacre with the hierarchical fibrillar organization of soft biological tissues. This approach fabricates anisotropic hydrogels with significantly improved fatigue thresholds versus traditional methods.

**Figure 5:**
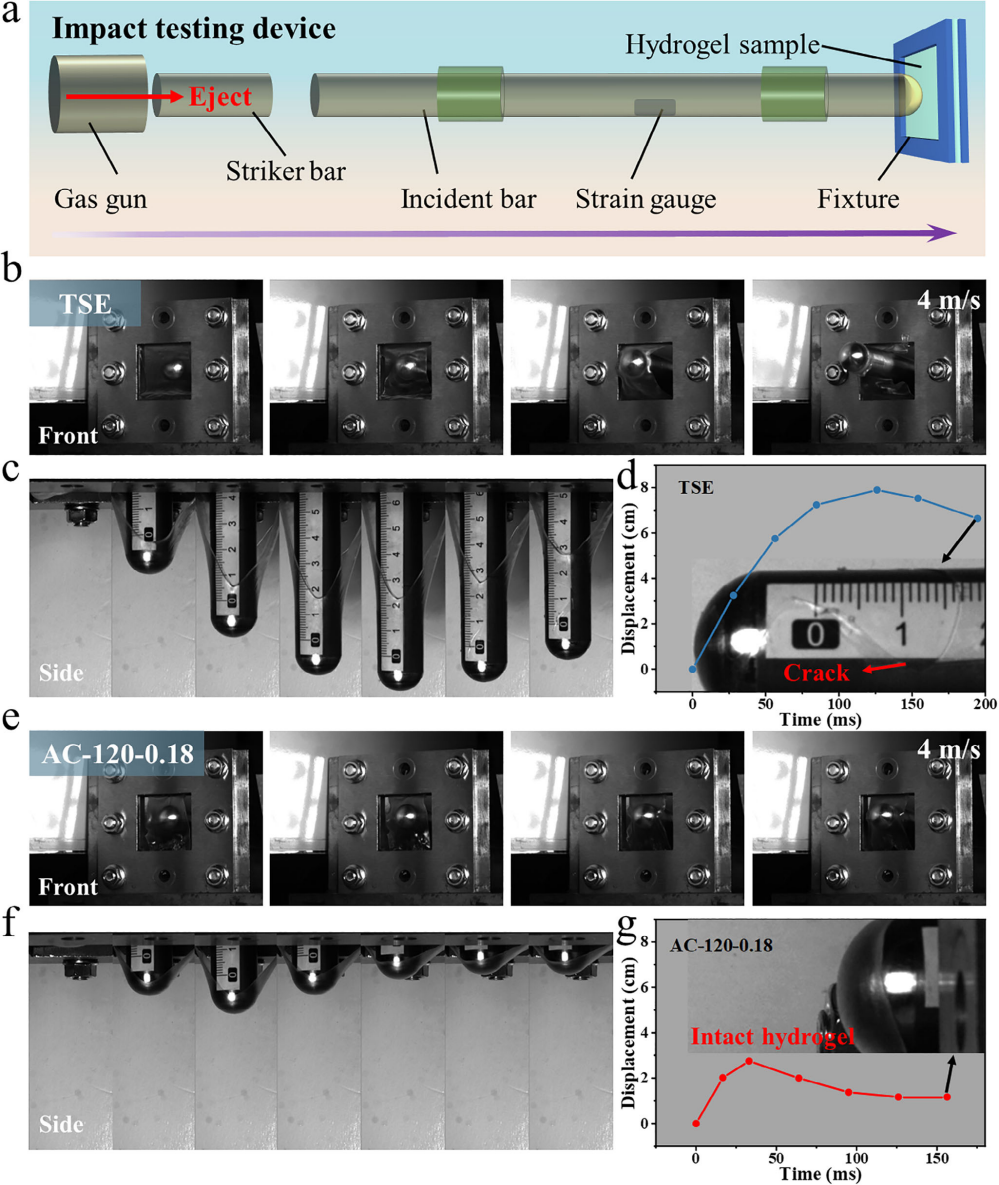
Anti‐impact properties of the anisotropic hydrogels. a) Schematic Split Hopkinson pressure bar system. High‐speed snapshot images showing b) TSE and e) AC‐120‐0.18 impacted by the incident bar from front and side. c,f) Representative side images, and d,g) corresponding displacement‐time curves for TSE and AC‐120‐0.18, respectively.

As is well known, hydrogels are prospective candidates for biomedical load‐bearing components, e.g., artificial tendon and ligament. In such cases, excellent impact resistance is required for hydrogels. However, the strength and elastic modulus of many traditional hydrogels are so strong, which often lead to material fracture in high‐energy impact applications. **Figure** **5**a shows a Split Hopkinson pressure bar (SHPB) system, which is a high strain rate testing machine for dynamic materials properties,^[^ ^38^, ^39^ ^]^ used to evaluate the impact resistance of TSE and AC‐120‐0.18. The striker bar is ejected from the gas gun and push the incident bar to move forward with a high velocity to impact directly on the hydrogel sample. Deformation and failure can occur depending on the hydrogel's impact resistance. Figure 5b,c displays the images of TSE during impact testing. At an impact velocity (*v*_i_) of 4 m s^−1^, TSE exhibited large deformation (Video S1, Supporting Information) represented by a displacement‐time curve with a maximum displacement of 7.9 cm at 123 ms. A crack started at 154 ms and spread quickly. TSE fractured at 195 ms (Figure 5d). By contrast, at the same *v*_i_ of 4 m s^−1^, AC‐120‐0.18 showed a small deformation, with a maximum displacement of only 2.75 cm at 33 ms (Figure 5e–g). Notably, AC‐120‐0.18 kept its structure integrity and effectively rebounded the incident bar during the impact test (Video S2, Supporting Information), which indicated that the high strength and high modulus of AC‐120‐0.18 could resist the transient high‐speed impact, and effectively absorbed a large amount of external energy. The excellent impact resistance of AC‐120‐0.18 is attributed to the multiscale synergistic mechanism. At the molecular level, the dynamic hydrogen‐bonding network converts impact kinetic energy into thermal energy through reversible bond‐breaking and reformation processes, while the highly oriented entangled polymer network effectively mitigates stress wave propagation via viscoelastic responses. Microstructurally, the aligned lamellar fibrous architecture not only dissipates energy through mechanisms such as fiber pull‐out and interfacial slippage, but its unique geometric alignment also enables directional transmission of stress waves. This synergistic interaction between molecular and microstructural hierarchies allows the material to simultaneously maintain structural integrity and achieve effective rebounding of the impact bar.

Therefore, to conclude, the hydrogels’ superior mechanical performance including high strength, toughness, fatigue and impact resistance enables them to withstand complex mechanical loads in dynamic physiological environments. Moreover, the tunable anisotropic architecture closely replicates the directional mechanical behavior and functional characteristics of native tissues such as cartilage and tendons. Importantly, the use of physical crosslinking, without the incorporation of toxic chemical crosslinkers, ensures excellent biocompatibility and thus supports their suitability for long‐term in vivo applications. Despite these promising features, several challenges remain, such as the slow degradation rate of PVA may not coincide with the desired timeline for tissue regeneration, which could impede full functional integration. Nevertheless, calendering‐enabled strong anisotropic hydrogels still show great advantages in implantable load‐bearing biomaterials due to their excellent mechanical properties and high water content.

## 3 Conclusion

Biomimetic anisotropic hydrogels with a fiber laminate structure were first developed in this work by a facile organogel‐assisted calendering strategy. This strategy is a breakthrough on the limitation of existing methods (e.g., freeze‐casting and pre‐stretching) by adjusting the polymer conformation and crystallization, hence improving substantially the mechanical properties of the hydrogels. The tensile strength, elastic modulus, toughness, fracture energy, and fatigue threshold of AC‐120‐0.18 could reach as high as 41.0 ± 6.4 MPa, 67.0 ± 5.1 MPa, 16.36 ± 4.26 MJ m^−3^, 62.20 ± 8.55 kJ m^−2^ and 2170 J m^−2^, respectively; and it also exhibits excellent anti‐fatigue, anti‐swelling and anti‐impact properties. Importantly, this new and simple approach will fulfill the urgent demand for fabrication of large‐size hydrogels, and the calendering‐enabled strong yet tough hydrogels have great potential for implantable load‐bearing biomaterials applications.

## 4 Experimental Section

The experimental section is shown in the Supporting Information.

## Conflict of Interest

The authors declare no conflict of interest.

## Supporting information



Supporting Information

Supplemental Video 1

Supplemental Video 2

## Data Availability

The data that support the findings of this study are available from the corresponding author upon reasonable request.
