# Supplementary material for: Highly Oriented Bio‐Mimetic Hydrogels by Calendering
Source: Adv Sci (Weinh). 2025 Jun 19;12(30):e04778. doi: 10.1002/advs.202504778 (PMC12376593; doi:10.1002/advs.202504778)
Supplement: Supplementary file 1 — Supporting Information [file ADVS-12-e04778-s002.docx]

Supporting Information

**Highly Oriented Bio-Mimetic Hydrogels by Calendering**

Zhanqi Liu^a^, Yuqing Wang^a^, Haidi Wu^a^, Huamin Li^a^, Longcheng Tang^b^, Guo Wang^c^, Daxin Zhang^c^, Jianping Yin^c^, Yinggang Miao^c^, Yongqian Shi^d^, Pingan Song^e^, An Xie^a^, Xuewu Huang^f^, Wancheng Gu ^a^, Yiu Wing Mai^g^_,_ Jiefeng Gao^*^^a^

^a^ *School of Chemistry and Chemical Engineering, Yangzhou University, No 180, Road Siwangting, Yangzhou, Jiangsu, 225002, China*

^b^ *Key Laboratory of Organosilicon Chemistry and Material Technology of Ministry of Education, College of Material, Chemistry and Chemical Engineering, Hangzhou Normal University, Hangzhou, 311121, China*

^c^ *Shanxi Key Laboratory of Impact Dynamics and its Engineering Application, School of Aeronautics, Northwestern Polytechnical University, Xi’an, 710072, China*

^d^ *College of Environment and Safety Engineering, Fuzhou University, Fuzhou 350116, China*

^e^ *Centre for Future Materials, University of Southern Queensland, Springfield Campus, QLD 4300, Australia*

^f^ *Testing Center, Yangzhou University, Yangzhou, Jiangsu, 225002, China*

^g^ *Department of Mechanical Engineering, The Hong Kong Polytechnic University, Hung Hom, Kowloon, Hong Kong, 999077, China*

*Corresponding author: E-mail address: jfgao@yzu.edu.cn

**Experimental Section**

**Materials and chemicals**

PVA power (Aladdin), glycerol (Aladdin), glutaraldehyde (Aladdin), and hydrochloric acid (Sinopharm Chemical Co., Ltd.) were used without further purification.

**The preparation of different hydrogels**

The preparation of anisotropic polyvinyl alcohol (PVA) hydrogels was as follows: A certain mass of PVA powder was dissolved in deionized water and stirred at 100 ℃ for 5 h to obtain a homogeneous PVA solution (15 wt.%), and then the solution was poured in a home-made polytetrafluoron mold and immersed in glycerol. After the solvent exchange for 48 h, PVA/glycerol organogel was obtained, and cut into rectangular pieces for calendering (Laboratory desk type calender, BD-8818 (2 inches, Dongguan Baoding Precision Instrument Co., LTD, China).

The PVA/glycerol organogel was thinned down and rolled for 15 min under a strong temperature-shearing field. The temperature for calendering was set at ambient (R), 70, 120 and 150 °C, and the roll gap was controlled at 0.6, 0.3, and 0.18 mm, respectively, with an average roll speed of 25 rad min^-1^. Notably, at excessively low temperatures (R and 70°C), the thermoplasticity of the glycerol organogel is insufficient to achieve an oriented structure, while at excessively high temperatures (150°C), the organogel dissolves under high pressure. Through experimental exploration, the optimal processing temperature was determined to be 120°C. At 120°C, the organogel could not form an anisotropic structure when the roll gap was more than 0.6 mm, whereas a roll gap of less than 0.18 mm resulted in organogel fracture. The experimental results showed that the roll gap for obtaining a complete anisotropic structure ranges from 0.18 to 0.6 mm.

Subsequently, The calendered organogels were immersed into deionized water to obtain the hydrogels, during which the glycerol was completely replaced by water. PVA hydrogels as “control” samples were also prepared by the two-step solvent exchange (TSE) without calendering.

**Mechanical tests**

For tensile characterization, hydrogel specimens were sectioned into dumbbells, their thickness was measured by a digital micrometer, and were stretched at a speed of 50 mm/min on a universal testing machine (Instron 3367, Instron, USA). The elastic modulus was calculated from the slope of the initial linear region of the stress-strain curve, and toughness was defined as the area under the stress-strain curve (J m^-3^) of the hydrogel sample at fracture. Cyclic loading-unloading tests were conducted at a constant rate of 50 mm/min on a customized mechanical stretcher (FULETEST, China). The fracture energy was measured by the pure shear test with an Instron 3367 testing machine. Briefly, force-displacement curves of notched and unnotched samples with the same initial dimensions were measured under the same test conditions. The fracture energy can be calculated by: *Γ*= *U*(*L_c_*)/*A*, where *A* is the cross-sectional area of the sample, and the critical distance where the notch turned into a running crack is represented by *L_c_*, and the work done to stretch an unnotched sample to *L*_c_ is defined as *U*(*L_c_*)*.*

**Fatigue tests**

A single-notch method was employed to assess the fatigue resistance of the hydrogels. To preserve the water content of the hydrogels, all the tests were performed in a water bath. Notched and unnotched samples with identical rectangular shapes were tested. Notched samples with crack lengths less than 1/5 of the width were subjected to cyclic tensile testing at a loading rate of 900 mm/min and a strain of λ allowing no relaxation using a customized mechanical stretcher (FULETEST, China. Meanwhile, a digital camera (AF4915ZTL, Dino-Lite) was used to record *in-situ* the fatigue crack growth of the hydrogel samples. By applying the same tensile strain ($\text{λ}$) to the unnotched sample, the *N*^th^ cyclic strain energy, denoted as *W*, is calculated by:

$$\text{ }\text{W}\text{(}\text{λ}\text{, }\text{N}\text{)=}\int_{\text{1}}^{\text{λ}} \text{S}\text{d}\text{λ} (S1)$$

where *S* is stress, and the energy release rate (*G*) is determined from:

$$\text{ }\text{G}\left( \text{λ}\text{, }\text{N} \right)\text{=2}\text{k }\left( \text{λ} \right)\text{×}\text{c }\left( \text{N} \right)\text{×}\text{W }\text{(}\text{λ}\text{, }\text{N}\text{) }\text{ }\text{ (}\text{S2) }$$

where $\text{k}\text{=3/}$*λ* is calculated empirically based on the strain variation, and *c* refers to the crack length after the *N*^th^ cycle. The fatigue threshold is determined by the intercept of the linear extrapolation of the data of crack extension per cycle (d*c*/d*N*) versus *G* to the abscissa.

**Morphology** **characterization**

The hydrogel samples were frozen and broken in liquid nitrogen, and then freeze-dried for 48 h. The obtained aerogels were examined using a scanning electron microscope (SEM, Zeiss Supra55, Germany) after gold spraying of the fracture surface. Atomic force microscope (SPM-9700HT, SHIMADZU, Japan) and optical microscope (Bresser 52-01005, German) were used to observe the surface morphology of different hydrogels.

**SAXS and WAXS measurements**

SAXS and WAXS measurements were conducted using the NanoSTAR (Bruker AXS, Germany) instrument equipped with X-rays with a wavelength of 0.154 nm, an operating voltage of 50 kV, and a current of 0.6 mA. The sample-to-detector distance was 1045 mm for SAXS and 60 mm for WAXS, and the exposure time was set as 600 s. The scattering range of *~~q~~* was 0.007 - 0.123 Å^-1^ for the SAXS measurements, and the WAXS profiles were collected in the 2θ range of 5° to 35°, and the scattering range was 0.21-2.45 Å^-1^. Based on the one-dimensional (1D) scattering curve of corrected scattering intensity (*Iq^2^*) *versus* scattering vector (*q*) from 2D SAXS patterns, the average distance between crystalline domains of PVA hydrogels was calculated using the following Bragg expression:

$$\text{L}\text{=}\frac{\text{2π}}{\text{q}_{\text{max}}\text{ }} (S3)$$

where $q_{max}$ is the critical vector corresponding to the highest peak intensity. From the 1D scattering curve of intensity *versus* diffraction angle (2θ) of WAXS patterns, the average size of the crystalline domains (*D*) of PVA hydrogels was calculated based on Scherrer's equation below:

*D* = *kλ*/(*β*cos*θ*) (S4)

where *k, λ, β, θ* are the dimensionless shape factor, the wavelength of X-ray diffraction, the full width at half maximum of the peak, and Bragg angle, respectively. We assume the shape of the crystalline domains of PVA hydrogel is approximately spherical, and thus *k* is set as 1. The orientation degree (*Π* ) from the azimuthal-integrated intensity distribution curves of the WAXS patterns can be estimated using the following empirical equation:

*Π* = (180$-$FWHM) /180 (S5)

where FWHM is the half-height width of the azimuthal intensity distribution curve.

**Water content**

All the hydrogel samples were dried until a constant mass was reached. The mass of the hydrogel samples in swollen and dry states were defined as *m*_s_ and *m*_d_, respectively. Water content ($\text{W}_{\text{C}}$) of the hydrogels is calculated by:

$\text{W}_{\text{C}}\text{=}\frac{\text{m}_{\text{s}}\text{-}\text{m}_{\text{d}}}{\text{m}_{\text{s}}}\text{×100\%}$ (S6)

**DSC measurement**

Differential scanning calorimetry (DSC 8500, Perkin Elme, USA) was carried out to identify the crystallinity of the dry hydrogel sample. To avoid additional crystallization during drying, we crosslinked the PVA chains to fix the amorphous domains using acidic glutaraldehyde solution (consisting of 5 mL glutaraldehyde, 0.5 mL concentrated hydrochloric acid, and 105 mL deionized water), and then soaked the sample in deionized water to remove the mixed solvent. Finally, the sample was dried in an oven at 37 °C for 3 d to obtain the dry hydrogel sample. In the DSC measurement, the N_2_ flow rate was set to 30 mL/min and the sample was heated from 50°C to 250°C at a rate of 20°C/min. $\text{H}_{\text{crystalline}}$is the enthalpy of melting in the crystalline region per unit mass of the sample. Therefore, the crystallinity of dry hydrogel ($\text{X}_{\text{dry}}$) sample was calculated by:

$\text{ X}_{\text{dry}}\text{=}\frac{\text{H}_{\text{crystalline}}}{\text{H}_{\text{crystalline}}^{\text{0}}\text{ }}\text{×100\%}$ (S7)

where $\text{H}_{\text{crystalline}}^{\text{0}}$ is the enthalpy of fusion for 100 wt.% crystalline PVA (138.6 J/g), the corresponding crystallinity of the swollen sample can be calculated by:

*X*_swollen_ *= X*_dry_ (1−$\text{W}_{\text{C}}$). (S8)

**Transmittance characterization**

An UV-visible-near infrared absorption spectrometer (Cary 5000, Varian, USA) was used to evaluate the light transmittance of different hydrogels with the wavelength ranging from 400 to 800 nm.

**ATR-FTIR measurement**

Attenuated total reflection Fourier transform infrared (ATR-FTIR) spectra were obtained by an infrared spectrometer (Cary610/670, Varian, USA) to characterize the vibration of functional groups of hydrogels in the wavenumber range of 4000-500 cm^-1^.

**Rheological measurement**

The circular hydrogel samples (a diameter of 12 mm) were measured using a DHR rheometer (TA, USA). During the experiment, the temperature was kept at 25 °C.

**Impact tests**

Impact tests were performed using a modified Split-Hopkinson Pressure Bar (SHPB) system, which consisted of a gas gun, a striker bar, an incident bar, one strain gauge, a data acquisition system, and a high-speed camera.

**Supplementary Figures**


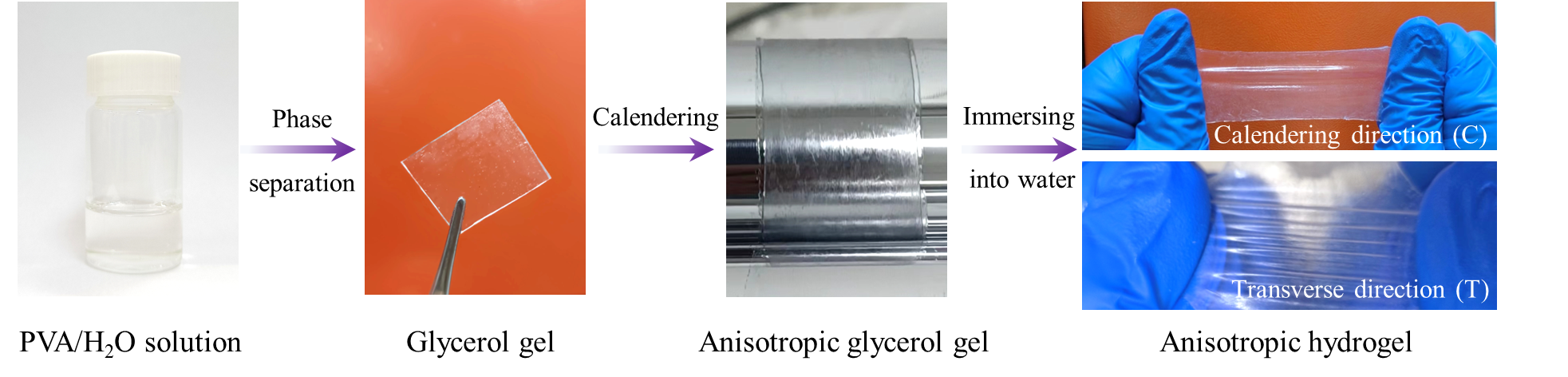


**Figure S1.** Photographs for fabrication of anisotropic hydrogels via the strategy of organogel-assisted calendering.

**Figure S2.** UV-vis transmittance spectra in the wavelength of 400-800 nm for different hydrogels.


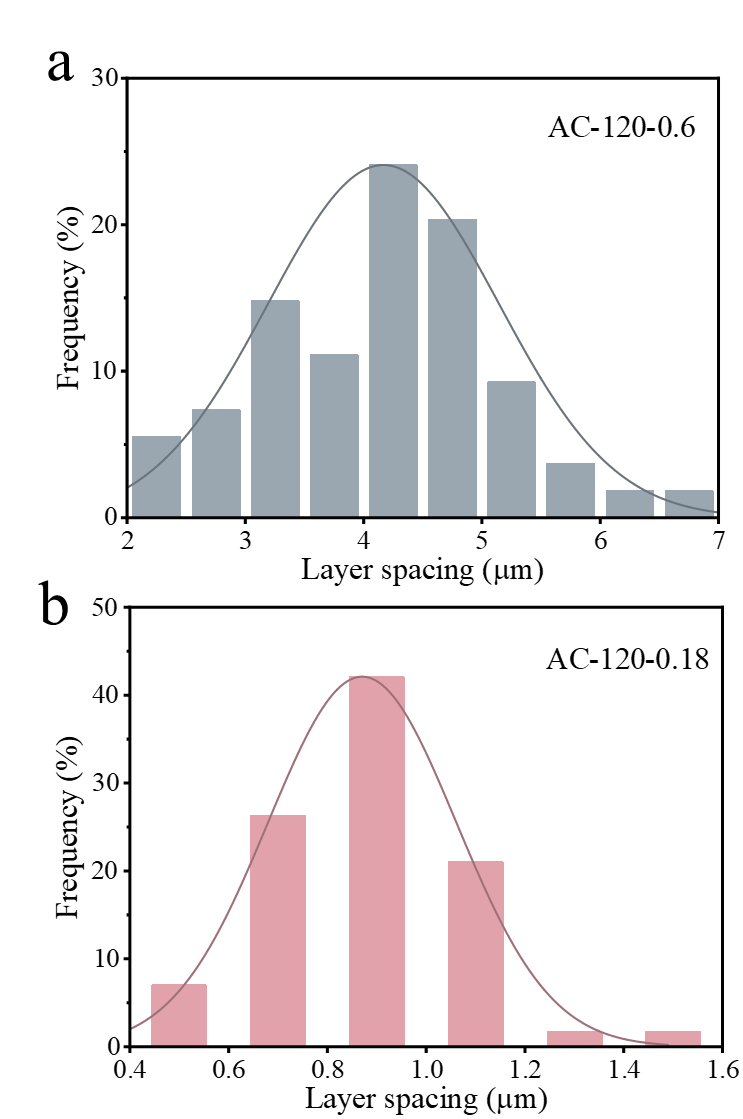


**Figure S3.** Histogram of fibril layer spacing distribution for a) TSE and b) AC-120-0.18.


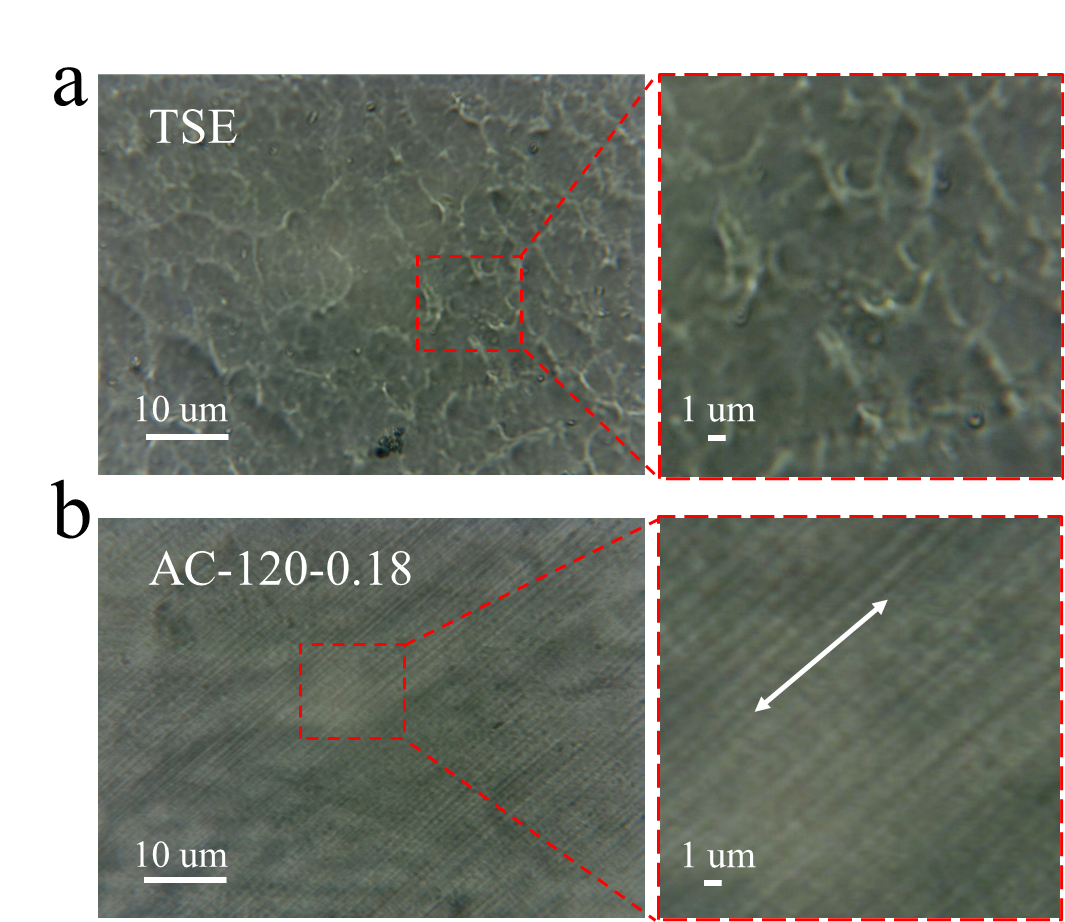


**Figure S4.** Optical microscope images of a) TSE and b) AC-120-0.18


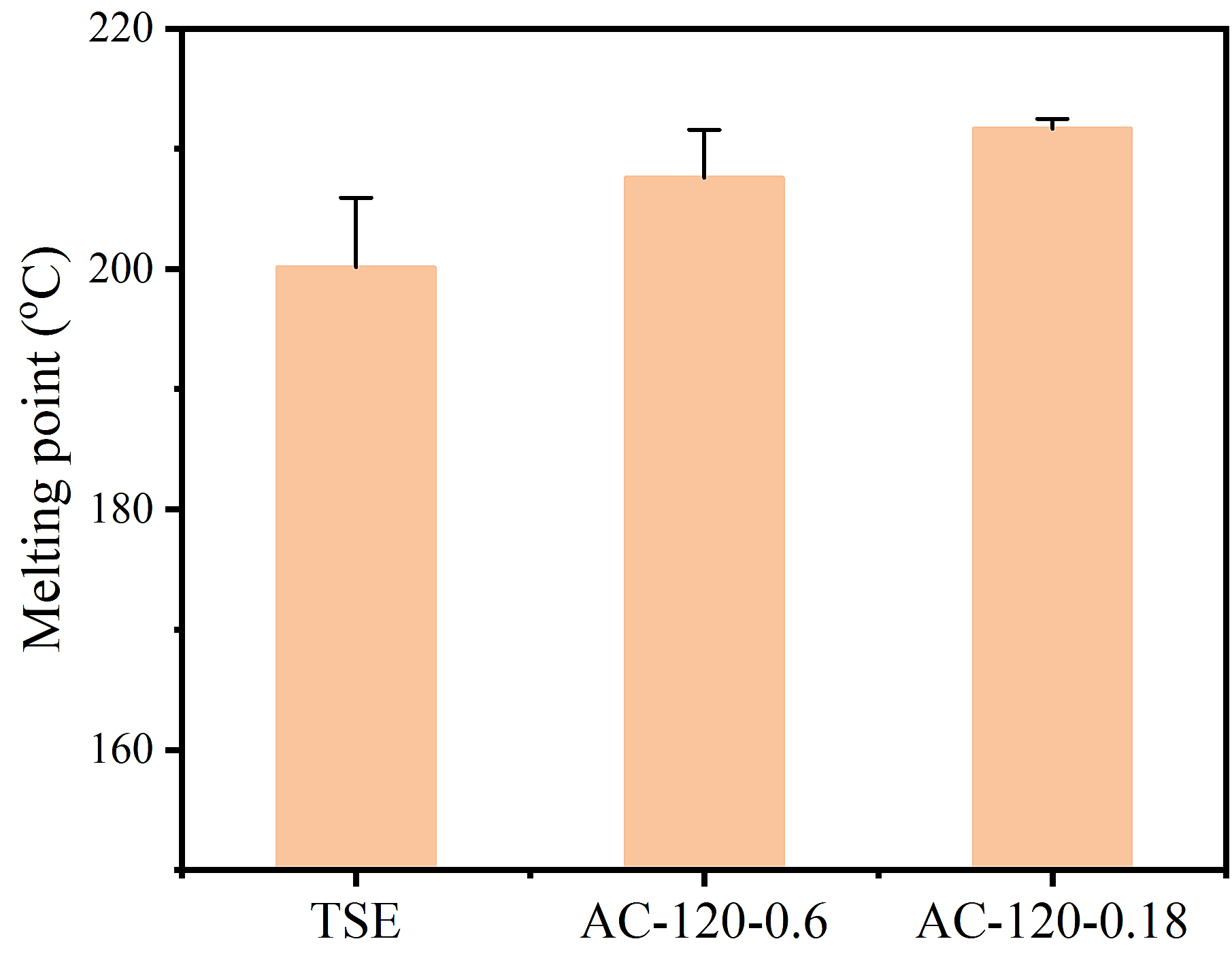


**Figure S5.** Summary of melting points for different hydrogels.


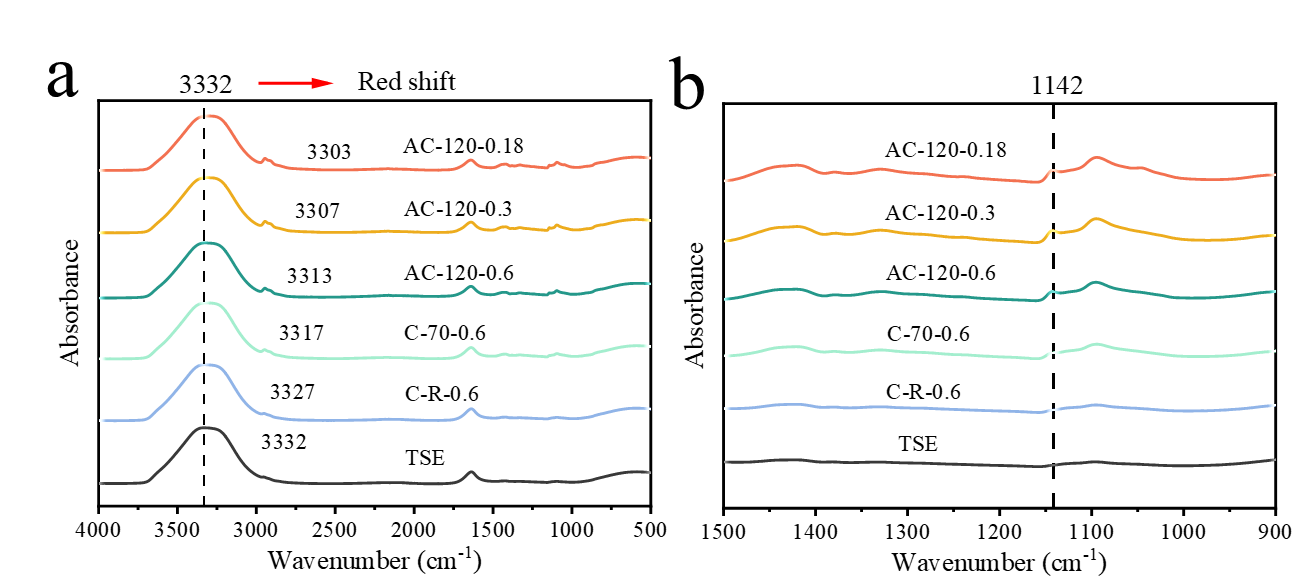


**Figure S6.** a) ATR-FTIR spectra of TSE, C-R-0.6, C-70-0.6, AC-120-0.6, AC-120-0.3 and AC-120-0.18. b) Enlarged spectra of the stretching variations of C-O (900-1500 cm^-1^) of different hydrogels.


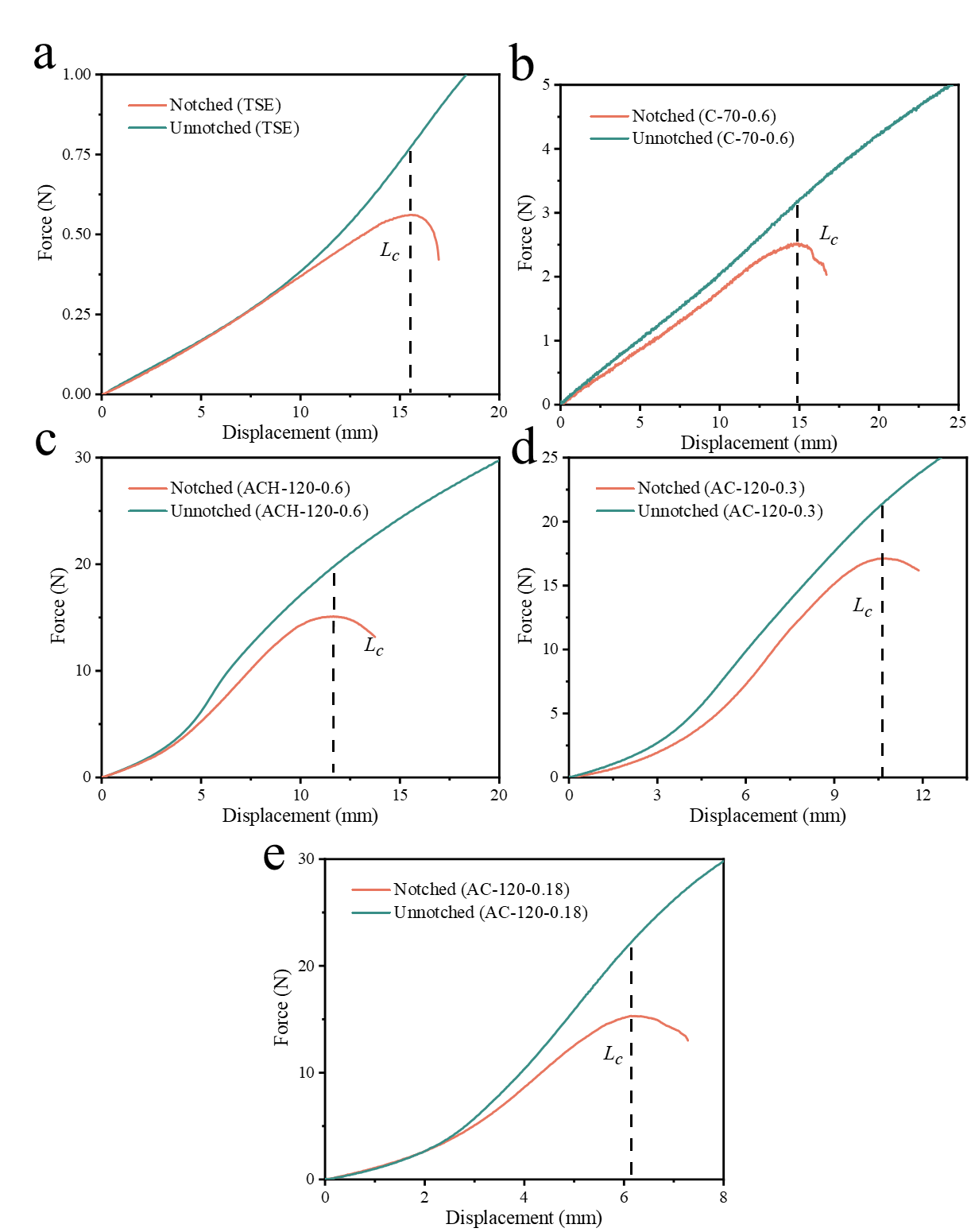


**Figure S7.** The force-displacement curves of unnotched and notched hydrogels for a) TSE, b) C-70-0.6, c) AC-120-0.6, d) AC-120-0.3 and e) AC-120-0.18. *L_c_* is displacement of unnotched sample when crack extension first occurs.

**
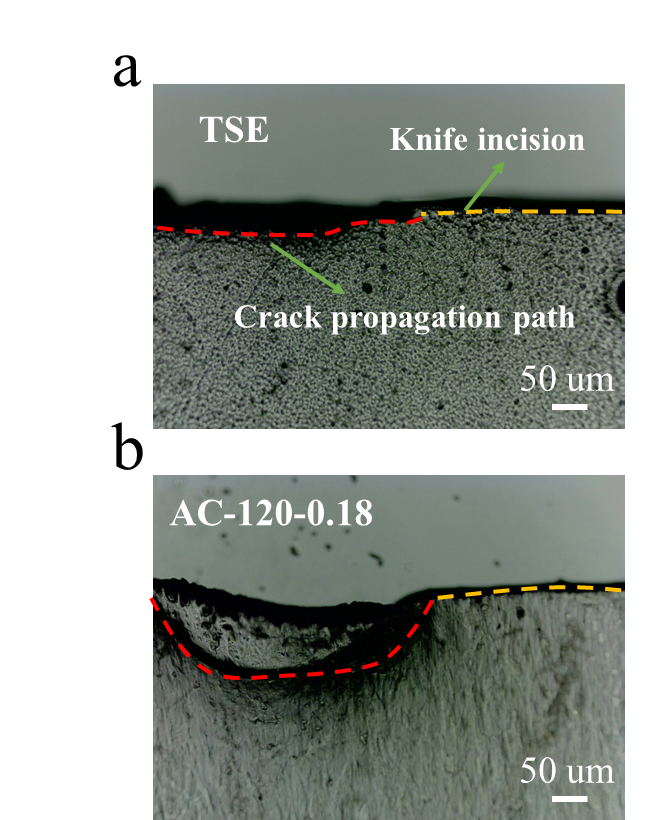
**

**Figure S8.** Optical microscope images of crack propagation of a) TSE and b) AC-120-0.18. The yellow dotted line represents the notch for fracture energy test, while the red dotted line represents the initial crack propagation path.


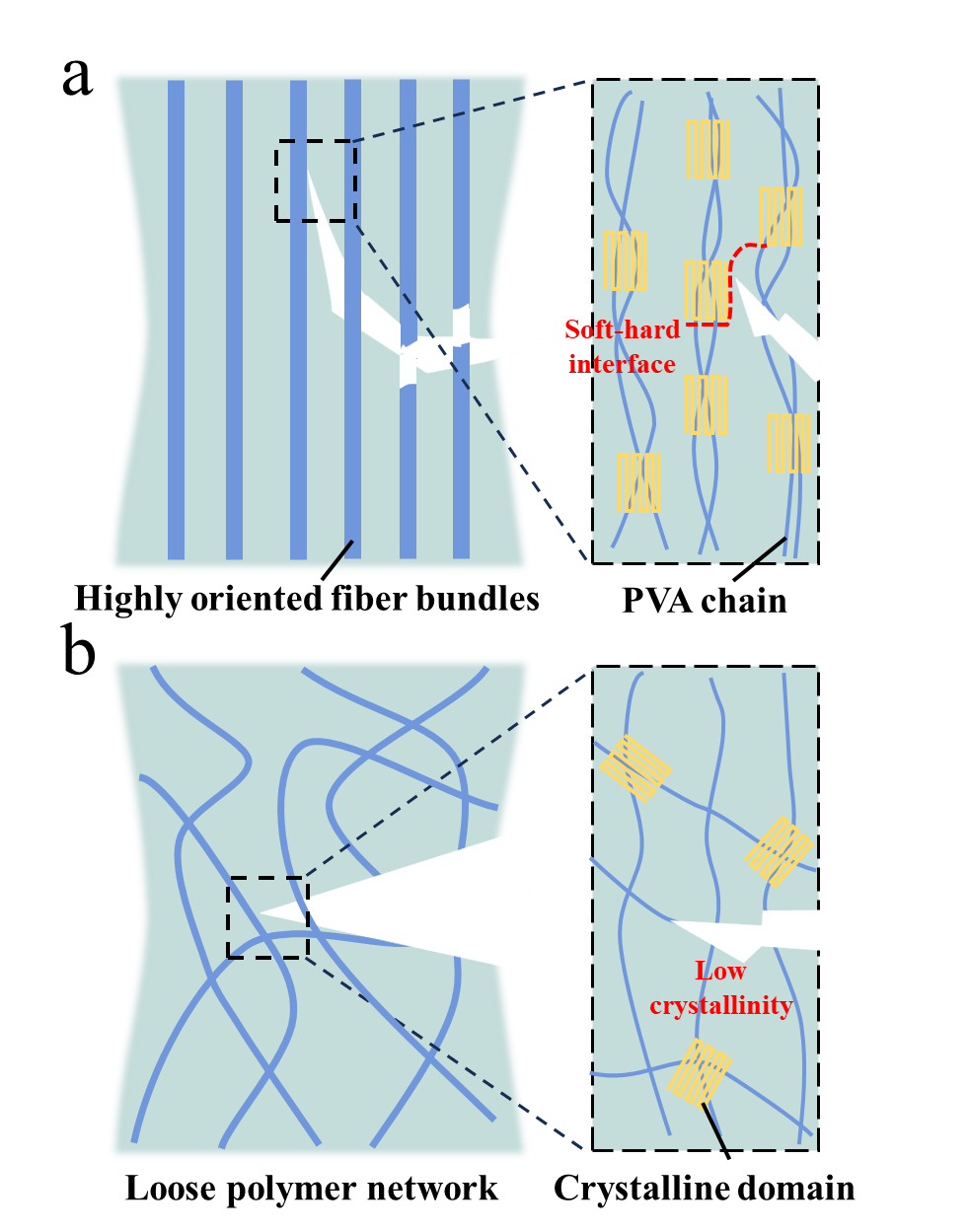


**Figure S9.** Schematic diagram of fracture mechanism for a) AC-120-0.18 and b) TSE.


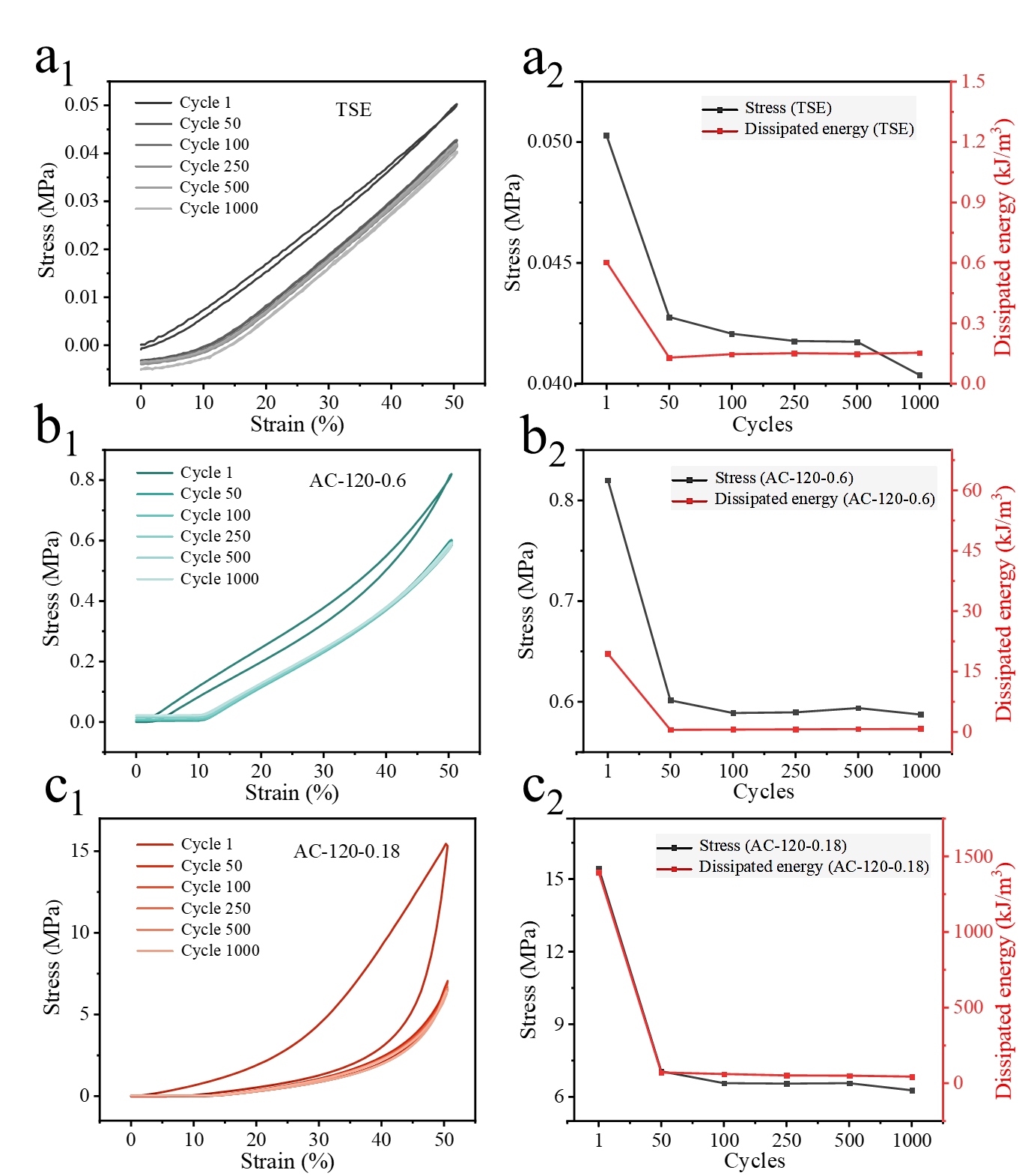


**Figure S10.** Stress-strain curves of a_1_) TSE, b_1_) AC-120-0.6 and c_1_) AC-120-0.18 with 1000 successive loading-unloading cycles under an applied strain of 50%, and a_2_), b_2_) and c_2_) their corresponding stress and dissipated energy during various loading-unloading cycles.


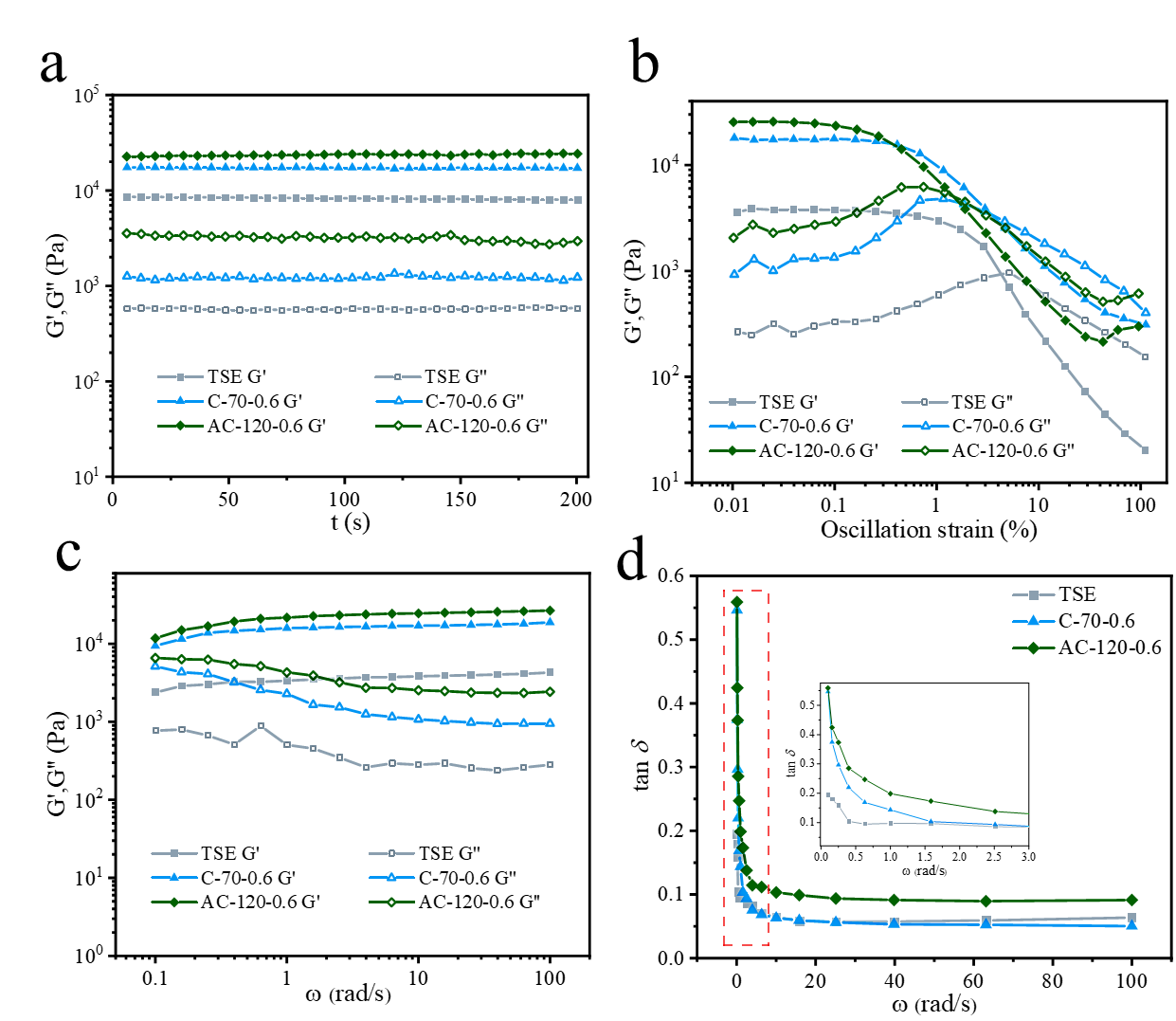


**Figure S11.** Rheological performance of different hydrogels. Storage modulus (*G*′) and loss modulus (G″) *versus* a) oscillation time, b) oscillation strain (%), and c) angular frequency. d) The loss factor (tan δ) *versus* angular frequency.


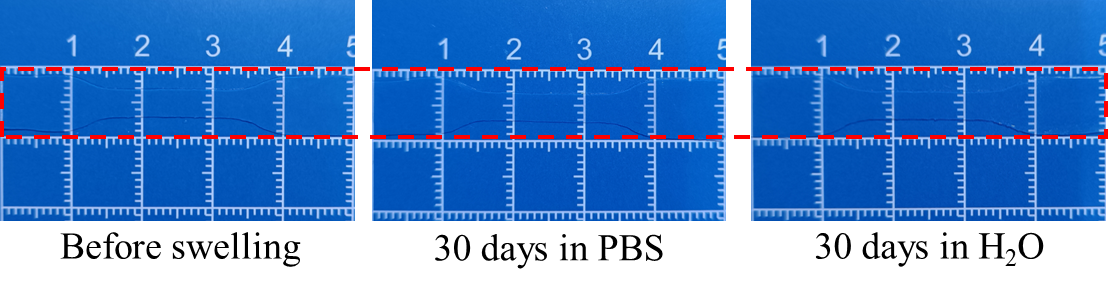


**Figure S12.** Photographs of AC-120-0.18 before and after swelling in PBS and water for 30 d.

**Figure S13.** Summary of fatigue threshold (Γ_0_) for different hydrogels.

**Supplementary Videos**

**Video S1.** Impact test of TSE

**Video S2.** Impact test of AC-120-0.18

**Table S1.** Comparison of mechanical properties of our anisotropic hydrogels with other hydrogels.

| **References** | **Materials** | **Structure/Method** | **Tensile strength (MPa)** | **Elastic modulus (MPa)** | **Toughness (MJ.m^-3^)** | **Fatigue threshold (J m^-2^)** | **Water/**  **solvent content (wt.%)** |
| --- | --- | --- | --- | --- | --- | --- | --- |
| ***This work*** | ***PVA hydrogel*** | ***Anisotropy/***  ***Calendering*** | ***12.6***  ***21.9***  ***41.0*** | ***2.2***  ***15.8***  ***67.0*** | ***46.2***  ***30.4***  ***16.4*** | ***1096***  ***1532***  ***2089*** | ***67***  ***65***  ***62*** |
| [1] | Cellulose hydrogel | Anisotropy/  Pre-stretching | 7.98 | 37.93 | 1.08 | NA | 85 |
| [2] | PVA+CNF hydrogel | Anisotropy/  Pre-stretching | 40 | 30 | 60 | NA | NA |
| [3] | Alginate hydrogel  Cellulose hydrogel | Anisotropy/Drying in confined condition (DCC) | 19.8  39.33  53.55 | 367.35  150.67  342.44 | 7.04  32.04  16.44 | NA | 56  58  58 |
| [4] | PVA hydrogel | Anisotropy/Mechanical training | 5.2 | 0.2 | NA | 1250 | 84 |
| [5] | PVA+Mxene hydrogel | Anisotropy/ Mechanical stretching-salting out | 16.57 | 8.58 | 39.23 | NA | 57 |
| [6] | Cellulose hydrogel | Anisotropy | 8.66  6.47 | 18.28  15.29 | NA | NA | 72  44 |
| [7] | PVA hydrogel | Anisotropy/ Freeze-casting and annealing | 2.5/18* | ~0.7/8* | NA | 1340/2740 | 90.8  82 |
| [8] | PVA+CNF+PEDOT:PSS hydrogel | Anisotropy/  Pre-stretching | 3.71 | 1.1 | 9.86 | NA | 72.5 |
| [9] | PVA+CNT hydrogel | Anisotropy/Freeze-casting | 4.5 | NA | NA | 1467 | 79.5 |
| [10] | Cellulose hydrogel | Anisotropy/  Pre-stretching | 47 | 140 | 20 | NA | 68 |
| [11] | PAAm hydrogel/ elastomer | Entanglements | 0.39/3.2 | ~0.1/1.2* | NA | 200/240 | NA |
| [12] | PVA hydrogel | Wet annealing | 5.11  11.19 | 0.76  2.67 | 51.66  82.28 | 557  1233 | ~67.5*  ~56* |
| [13] | PVA hydrogel | Dry-annealed | ~4/6/9* | ~1/3/10* | NA | ~750/900/1* | 65.8/62.2/59.9 |
| [14] | PVA hydrogel | Salting out | 25.2 | 52.3 | NA | NA | NA |
| [15] | PVA+urea hydrogel | Hydrogen bond | 23.8 | 11.28 | NA | NA | 45.2 |
| [16] | PVA hydrogel | Salting-out | 26.72 | ~5.5* | 55.21 |  | ~53 |
| [17] | PAAc/PAAm hydrogel | Dual-crosslinked | 10 | 17 | 26.47 | NA | 50 |
| [18] | Poly(MAA-co-OEGMA) hydrogel | π–π interactions | 3.7 | 10 | 6.6 | NA | NA |
| [19] | PVA+ANF hydrogel | Hydrogen bond | 5 | 9.1 | NA | NA | 70 |
| [20] | PVA hydrogel | Solvent-exchange | 5 | 1 | 15 | NA | 72 |
| [21] | PVA+SNF (SiO_2_ nanofibers) hydrogel | Freeze-drying and  annealing | 7.84 | 13.71 | 9.9 | NA | NA |
| [22] | Cellulose hydrogel | Dual cross-linking and annealing | 7.2 | 5.4 | 5.6 | NA | NA |
| [23] | PVA+GO hydrogel | Bidirectional freeze-casting and compression-  annealing | 8.8 | ~9* | NA | 1567/1539 | ~82/78* |
| [24] | PAAM/polyprotein hydrogel | Polyprotein crosslinking | ~0.16* | ~0.019* | NA | 126 | NA |
| [25] | Polyacrylamide hydrogel | Single-network/ Solvent exchange | NA | 0.01127/0.02109 | NA | 20.5/64.5 | 78/69 |
| [26] | PAMPS/PAAM [hydrogels](https://www.sciencedirect.com/topics/engineering/double-network-hydrogel) | Double network (DN) | 0.7 | 0.11 | NA | 418 | NA |
| [27] | Fiber embedded in hydrogel matrix | Composite | 0.25 | 0.40135 | NA | 1290 | 70 |
| [28] | PVA hydrogel | Nanofibrous network | 3.5/8.4 | NA | NA | 600/770 | NA |
| [29] | Polyampholyte hydrogel (PA) | Ionic bond and covalent bond | ~1.3* | 0.14 | NA | 67.3/69.8/71.1 | 46.1/45.5/45.5 |
| [30] | PAAm+ANF hydrogel | Hydrogen bond/ covalent bond | ~0.07/0.125/0.175* | ~0.013/0.02/0.039* | NA | ~12.602/22.950/29.795* | NA |

**References**

[1] D. Ye, P. Yang, X. Lei, D. Zhang, L. Li, C. Chang, P. Sun and L. Zhang, *Chem. Mater.* **2018**, *30*, 5175-5183.

[2] L. Wu, Y. Kang, X. Shi, B. Yuezhen, M. Qu, J. Li and Z.-S. Wu, *ACS nano* **2023**, *17*, 13522-13532.

[3] M. T. I. Mredha, Y. Z. Guo, T. Nonoyama, T. Nakajima, T. Kurokawa and J. P. Gong, *Adv. Mater.* **2018**, *30*, 1704937.

[4] S. Lin, J. Liu, X. Liu and X. Zhao, *Proc. Natl. Acad. Sci. U.S.A.* **2019**, *116*, 10244-10249.

[5] B. Guo, Y. Wu, S. He, C. Wang, M. Yao, Q. Yu, X. Wu, C. Yu, M. Liu, L. Liang, Z. Zhao, Y. Qiu, F. Yao, H. Zhang and J. Li, *J. Mater. Chem. A* **2023**, *11*, 8038-8047.

[6] S. Zhou, K. Guo, D. Bukhvalov, W. Zhu, J. Wang, W. Sun and M. He, *J. Mater. Chem. A* **2021**, *9*, 5533-5541.

[7] X. Liang, G. Chen, S. Lin, J. Zhang, L. Wang, P. Zhang, Z. Wang, Z. Wang, Y. Lan, Q. Ge and J. Liu, *Adv. Mater.* **2021**, *33*, 2102011.

[8] N. Li, Q. Yu, S. Duan, Y. Du, X. Shi, X. Li, T. Jiao, Z. Qin and X. He, *Adv. Funct. Mater.* **2024**, *34*, 2309500.

[9] S. Han, Q. Wu, J. Zhu, J. Zhang, A. Chen, S. Su, J. Liu, J. Huang, X. Yang and L. Guan, *Mater. Horizons* **2023**, *10*, 1012-1019.

[10] M. T. I. Mredha, H. H. Le, P. Trtik, J. Cui and I. Jeon, *Mater. Horizons* **2019**, *6*, 1504-1511.

[11] J. Kim, G. Zhang, M. Shi and Z. Suo, *Science* **2021**, *374*, 212-216.

[12] Y. Wu, Y. Zhang, H. Wu, J. Wen, S. Zhang, W. Xing, H. Zhang, H. Xue, J. Gao and Y. Mai, *Adv. Mater.* **2023**, *35*, 2210624.

[13] S. Lin, X. Liu, J. Liu, H. Yuk, H.-C. Loh, G. A. Parada, C. Settens, J. Song, A. Masic, G. H. McKinley and X. Zhao, *Sci. Adv.* **2019**, *5*, eaau8528.

[14] L. Xu, Y. Qiao and D. Qiu, *Adv. Mater.* **2023**, *35*, 2209913.

[15] Y. Wu, Y. Shi and H. Wang, *Macromolecules* **2023**, *56*, 4491-4502.

[16] D. Liu, Y. Cao, P. Jiang, Y. Wang, Y. Lu, Z. Ji, X. Wang and W. Liu, *Small* **2023**, *19*, 2206819.

[17] P. Lin, S. Ma, X. Wang and F. Zhou, *Adv. Mater.* **2015**, *27*, 2054-2059.

[18] Z. Jiang, M. L. Tan, M. Taheri, Q. Yan, T. Tsuzuki, M. G. Gardiner, B. Diggle and L. A. Connal, *Angew. Chem. Int. Ed.* **2020**, *132*, 7115-7122.

[19] L. Xu, X. Zhao, C. Xu and N. A. Kotov, *Adv. Mater.* **2018**, *30*, 1703343.

[20] X. Yang, L. Xu, C. Wang, J. Wu, B. Zhu, X. Meng and D. Qiu, *Adv. Mater.* **2023**, *35*, 2303728.

[21] Y. Ma, J. Gong, Q. Li, X. Liu, C. Qiao, J. Zhang, S. Zhang and Z. Li, *Small* **2024**, *20*, 2310046.

[22] P. Wei, X. Yu, Y. Fang, L. Wang, H. Zhang, C. Zhu and J. Cai, *Small* **2023**, *19*, 2301204.

[23] X. Liang, G. Chen, S. Lin, J. Zhang, L. Wang, P. Zhang, Y. Lan and J. Liu, *Adv. Mater.* **2022**, *34*, 2107106.

[24] H. Lei, L. Dong, Y. Li, J. Zhang, H. Chen, J. Wu, Y. Zhang, Q. Fan, B. Xue and M. Qin, *Nat. Commun.* **2020**, *11*, 4032.

[25] E. Zhang, R. Bai, X. P. Morelle and Z. Suo, *Soft Matter* **2018**, *14*, 3563-3571.

[26] W. Zhang, X. Liu, J. Wang, J. Tang, J. Hu, T. Lu and Z. Suo, *Eng. Fract. Mech.* **2018**, *187*, 74-93.

[27] C. Xiang, Z. Wang, C. Yang, X. Yao, Y. Wang and Z. Suo, *Mater. Today* **2020**, *34*, 7-16.

[28] J. Ni, S. Lin, Z. Qin, D. Veysset, X. Liu, Y. Sun, A. J. Hsieh, R. Radovitzky, K. A. Nelson and X. Zhao, *Matter* **2021**, *4*, 1919-1934.

[29] X. Li, K. Cui, T. L. Sun, L. Meng, C. Yu, L. Li, C. Creton, T. Kurokawa and J. P. Gong, *Proc. Natl. Acad. Sci. U.S.A.* **2020**, *117*, 7606-7612.

[30] H. Xing, X. He, Y. Wang, X. Zhang, L. Li, Y. Wang, Z. Cheng, H. Wu, Q. Ge and X. Li, *Mater. Today* **2023**, *68*, 84-95.
